# Human adaptation and population differentiation in the light of ancient genomes

**DOI:** 10.1038/ncomms10775

**Published:** 2016-03-18

**Authors:** Felix M. Key, Qiaomei Fu, Frédéric Romagné, Michael Lachmann, Aida M. Andrés

**Affiliations:** 1 Department of Evolutionary Genetics, Max Planck Institute for Evolutionary Anthropology, 04103 Leipzig, Germany; 2 Key Laboratory of Vertebrate Evolution and Human Origins of Chinese Academy of Sciences, IVPP, CAS, Beijing 100044, China; 3 Department of Genetics, Harvard Medical School, Boston 02115, Massachusetts, USA; 4 Santa Fe Institute, Santa Fe, New Mexico 87501, USA

## Abstract

The influence of positive selection sweeps in human evolution is increasingly debated, although our ability to detect them is hampered by inherent uncertainties in the timing of past events. Ancient genomes provide snapshots of allele frequencies in the past and can help address this question. We combine modern and ancient genomic data in a simple statistic (DAnc) to time allele frequency changes, and investigate the role of drift and adaptation in population differentiation. Only 30% of the most strongly differentiated alleles between Africans and Eurasians changed in frequency during the colonization of Eurasia, but in Europe these alleles are enriched in genic and putatively functional alleles to an extent only compatible with local adaptation. Adaptive alleles—especially those associated with pigmentation—are mostly of hunter-gatherer origin, although lactose persistence arose in a haplotype present in farmers. These results provide evidence for a role of local adaptation in human population differentiation.

## Abstract (web summary)

Detecting the targets of positive selection in the human genome is challenging. Here, the authors combine modern and ancient genomes to show that alleles strongly differentiated between Africans and Europeans mediated local adaptation in European populations, and were mostly contributed by ancient hunter-gatherers.

## Graphical abstract

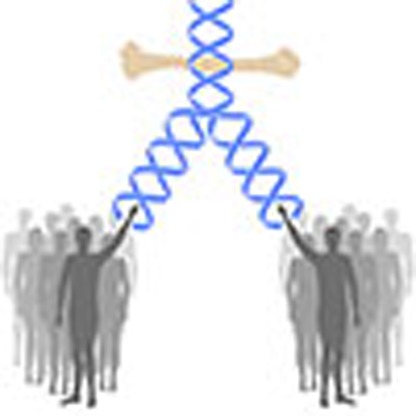

Genomes carry the footprint of their history, and inform us about the demographic and adaptive processes experienced by populations. For example, geographic distance between human populations correlates with their genetic allele frequency differentiation^1^,^2^, even when considering only the alleles with the strongest allele frequency differentiation^2^. This shows that neutral processes such as drift played a fundamental role in the (modest) genetic differentiation that exists among present-day human populations. However, it is also clear that populations have at least partially adapted to their local environment through natural selection, resulting in both genetic and phenotypic population differentiation^3^,^4^,^5^,^6^,^7^.

Establishing the contribution of neutral and adaptive processes to population genetic differentiation remains challenging. A strategy that has sometimes proven successful is to compare putatively neutral sites with those that are potentially non-neutral. For example, both putative targets of positive selection^3^ and the most differentiated alleles among human populations^2^,^8^ tend to be enriched in genic variants. This is consistent with local positive selection raising the frequency of adaptive genic alleles in certain populations^2^,^8^. Nevertheless, non-adaptive processes (for example, increased drift in genic regions due to purifying and background selection) also play a role. In fact, the excess of highly differentiated genic alleles can be recovered by simulations with background selection, leaving little evidence for the effect of local adaptation in population differentiation^2^. This suggests, perhaps counter-intuitively, that even the most extreme cases of population differentiation may be neutral. In addition, human adaptation might be largely polygenic^9^,^10^ resulting in modest population differentiation^11^,^12^,^13^. These views challenge the notion that large allele frequency differences between populations can be used to identify the targets of positive selection.

An important problem of existing approaches is that they are limited by the imperfect and indirect information that present-day genomes provide about the time of allele frequency changes. In humans it is, for example, difficult to precisely distinguish changes that happened during strong bottlenecks (for example, the out-of-Africa migration that substantially increased drift^14^) or in other periods (for example, during the subsequent colonization of territories outside of Africa, when populations likely adapted to novel environments). We address this question by exploiting the unprecedented availability of high-quality ancient human genomes, which provide snapshots of allele frequencies back in time.

A number of recent studies have used DNA of ancient Europeans to explore the evidence that positive selection raised the frequency of pre-ascertained single nucleotide polymorphisms (SNPs)^15^,^16^,^17^,^18^,^19^, showing that ancient DNA can enhance the identification of individual selective sweeps. We aim to investigate the influence of positive selection on population differentiation, rather than studying the trajectory of individual alleles. For this we use whole ancient genomes (up to 45,000 years old) to improve our inferences on the origin of allele frequency differences across human populations. Specifically, we integrate modern and ancient genetic information in a new statistic that helps disentangle the effects of drift and adaptation on rapid, geographically localized allele frequency changes. Incorporating this information provides clear evidence for positive selection raising the frequency of advantageous genic SNPs in Europe and contributing to strong population differentiation. These alleles include both previously identified targets of positive selection (for example, those involved in pigmentation and lactase tolerance) as well as many new alleles. We also show that these alleles were mostly contributed by ancient hunter gatherers, who resided in Europe thousands of years before the arrival of southern farmer groups.

## Results and Discussion

### Overview

Ancient genomes contain valuable information on the genetic make-up of ancestral populations, and combined with modern genomes hold the potential to improve the resolution of inferences on the timing of past allele frequency changes. Here we combine modern and ancient genomic information to study allele frequency differentiation and to explore the evidence for local adaptation in Eurasian human populations.

### Present-day population differentiation

**Figure 1:**
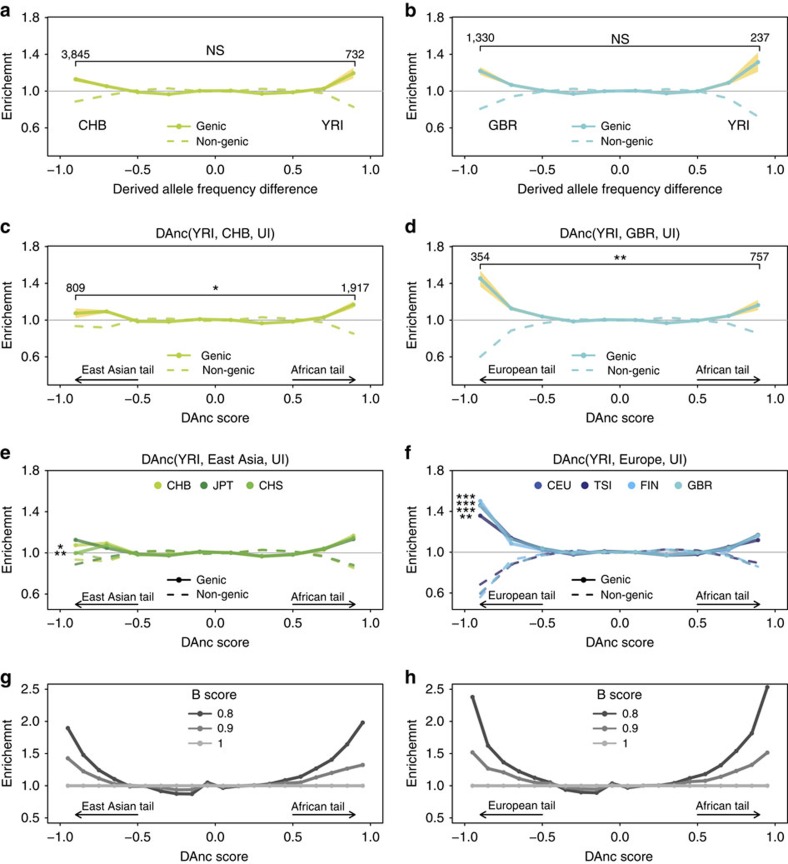
Analyses of population differentiation. (**a**,**b**) Genic enrichment among alleles in different bins of population differentiation between (**a**) CHB or (**b**) GBR and YRI. The bootstrap 95% confidence interval is shown in yellow, and the level of significance of the bias in genic enrichment when comparing the two tails is shown on top (*P< 0.05, **P<0.01 and ***P<0.001, and NS non-significant). The number of genic sites in each tail is on top of the tails. (**c**,**d**) Genic enrichment in alleles in different bins of the DAnc statistic (YRI, *P*_2_, Ust′-Ishim (UI)) using (**c**) CHB or (**d**) GBR as *P*_2_. The 95% confidence interval, the significance of the bias, and the number of genic alleles are shown as above. (**e**,**f**) Genic enrichment in alleles in different bins of the DAnc statistic when considering each East Asian (**e**) or European (**f**) population as *P*_2_, with significance of the bias between the tails indicated by asterisks (*<0.05, **<0.01 and ***<0.001). (**g**,**h**) Expectation of the genic enrichment across the DAnc distribution under non-adaptive forces, based on simulations with different strengths of background selection (measured by *B* scores) and with no background selection (*B*=1).

We first explored the genetic differentiation of present-day populations as done in previous approaches that considered lower number of SNPs^2^,^8^. For each SNP in the 1000 Genomes data set^20^, we compute the difference in derived allele frequency between pairs of populations, and then, for each bin in this distribution, we calculate the ratio of genic alleles versus all alleles (genic plus non-genic) normalized by the global ratio (following Coop *et al*.^2^, Methods). This shows a significant enrichment in genic SNPs among the most strongly differentiated alleles between any African and any European or East Asian population (Fig. 1a for CHB, Han Chinese in Beijing, China; Fig. 1b for GBR, British from England and Scotland; Supplementary Fig. 1 for other Eurasian populations). The same pattern emerges with other measures of population differentiation (F_ST_; Supplementary Fig. 2) and when significance is assessed through a weighted block jackknife^21^ to account for the presence of linkage disequilibrium (LD) in the tails (Supplementary Fig. 3). The pattern of genic enrichment is symmetric in both tails and (as shown by Coop *et al*.^2^) can be recovered by simulations with background selection (Supplementary Fig. 4). This is because background selection further increases the effect of drift during the out-of-Africa bottleneck, resulting in larger allele frequency changes in genic than in non-genic regions^22^.

Thus, after accounting for background selection, these analyses provide no evidence that local adaptation has driven allele frequency differentiation between Eurasian and African populations. Nevertheless, the approach is hampered by the fact that we cannot distinguish the many allele frequency changes that happened during the out-of-Africa bottleneck (largely due to drift) from the changes that occurred during the subsequent colonization of novel environments, which may have contributed to local adaptation. To elucidate the effect of both, ancient human genomes are particularly useful.

### Integrating ancestral genomes in population differentiation

**Figure 2:**
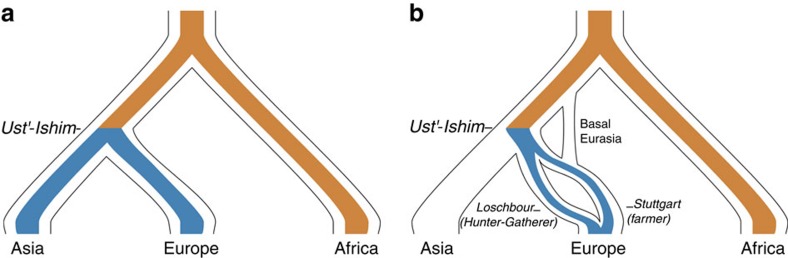
Tree of inferred relationships of present-day and ancient humans considered. (**a**) Including the ancient Ust′-Ishim sample, and (**b**) including the ancient Europeans samples. Colours indicate the branches where alleles in the European (blue) and African (orange) tails of the DAnc distribution most likely changed in allele frequency. For demographic parameters used see Methods and Supplementary Fig. 7.

We use the high-quality genome from Ust′-Ishim, a 45,000-year-old modern human whose remains were found in Siberia^23^. Ust′-Ishim has been inferred to belong to an early Eurasian population that shared the out-of-Africa bottleneck and the Neanderthal admixture with modern Eurasians^23^, and that diverged from the ancestors of Western and Eastern Eurasians before (or approximately simultaneously with) their split from each other (Ust′-Ishim is equally distant to East Asians and ancient Eurasians^23^; Fig. 2a). Thus, the Ust′-Ishim individual provides extremely valuable information about the genetic make-up of modern humans shortly after the out-of-Africa bottleneck. This sample yielded 42-fold genome-wide coverage^23^, so while no other comparable genome exists, the high-quality genotypes of the Ust′-Ishim genome can be used as a proxy for the genome-wide allele frequencies in its population. These are based on two chromosomes (so the frequency is 0, 0.5 or 1) and are obviously very noisy for any given SNP. Nevertheless, under the reasonable assumption that the individual is representative of its population (we have no evidence to the contrary), the two alleles should represent a random sample of the allele frequency distribution in Ust′-Ishim's population, which allows inferences at the genome scale.

We incorporate Ust′-Ishim in the analysis of present-day population differentiation with the *Differentiation with Ancestral* (DAnc) statistic, which is calculated per SNP:


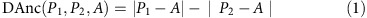


or


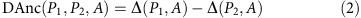


where *P*_*1*_ and *P*_*2*_ are the estimated derived allele frequency for present-day populations 1 and 2, and *A* for the ancestral population. The statistic can be extended to consider population-based ancestral information if available, but as discussed above here we use the Ust′-Ishim genotypes.

The DAnc statistic ranges from −1 to 1 and it is most informative in its extreme values. A DAnc of −1 or 1 can only be reached when *P*_1_ and *P*_2_ are 0 (or 1) for different alleles (a fixed difference between these two populations) and *A* is equal to one of them (here, Ust′-Ishim is homozygote for one of the two alleles). The range of intermediate DAnc values is obtained either with low-to-moderate allele frequency differentiation between *P*_1_ and *P*_2_ (regardless of *A*), or with *A* intermediate between *P*_1_ and *P*_2_ (regardless of *P*_1_ and *P*_2_), so intermediate values are not as informative. Alleles where *A* is highly differentiated from both *P*_1_ and *P*_2_, also fall in the intermediate DAnc range.

For example, given population *P*_1_ of African ancestry and population *P*_2_ of Eurasian ancestry DAnc (Africa, Eurasia, Ust′-Ishim), DAnc approximately 1 is compatible, ignoring uncertainty in allele frequency, with Ust′-Ishim being very similar to the Eurasian population, and both of them being very different from Africa (allele frequency changes inferred to have happened most likely over the orange part of Fig. 2a). For convenience we define the alleles with DAnc 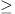
0.8 (alleles with |*P*_1_−*P*_2_| 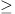
0.8 and Ust′-Ishim homozygote for the Eurasian allele) as the *African tail*. These sites are obviously strongly influenced by the out-of-Africa bottleneck, although they also include Africa-specific allele frequency changes. DAnc approximately −1 reflects the opposite scenario: Ust′-Ishim being very similar to Africa, both of them very different from Eurasia. This *European* or *East Asian tail* (DAnc 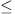
−0.8) contains the alleles that experienced a sharp increase in allele frequency on the Eurasian branch after Ust′-Ishim and thus after the out-of-Africa bottleneck (blue part of Fig. 2a). The inference is obviously noisy for each individual allele, but the Eurasian DAnc tails are presumably enriched for any putative target of strong, recent positive selection in Eurasian populations.

### DAnc for East Asian and European populations

We first used the DAnc statistic to investigate the allele frequency differentiation between Yoruba from Africa (Yoruba in Ibadan, Nigeria (YRI)) versus East Asia (CHB, Fig. 1c). There are 5,113 alleles in the tails of the DAnc (YRI, CHB, Ust′-Ishim) distribution, 69% falling in the African tail and 31% falling in the East Asian tail. This shows that the population differentiation in CHB is strongly influenced by the out-of-Africa bottleneck. When we investigate the enrichment of genic alleles across DAnc values, results largely agree with the two-population differentiation results above, showing an enrichment of genic alleles in the two tails of the DAnc distribution (Fig. 1c). The enrichment in genic alleles is slightly stronger in the African tail although not always significantly so (Fig. 1c), a pattern consistent across East Asian populations (Fig. 1e; Supplementary Figs 5 and 6) and expected with increased drift in genic regions during the out-of-Africa bottleneck. In fact, our simulations that combine realistic demographic models (Fig. 2a; Supplementary Fig. 7, Methods) with stronger background selection in genic loci recapitulate well these results (*B* score=0.9 in Fig. 1g).

We then compared Yoruba with Europe using GBR (Fig. 1d). The tails of this distribution contain less alleles than the tails of the East Asian one (1,910 SNPs), consistent with weaker drift in Europe compared with East Asia^24^. Of those alleles, 73% fall in the African tail and 27% in the European tail, again consistent with the influence of the out-of-Africa bottleneck. This analysis also shows an enrichment of genic alleles in both tails but, surprisingly, the enrichment is much stronger in the European tail. This results in a biased enrichment pattern, with the European tail being significantly more enriched in genic alleles than the African tail (Fig. 1d). The pattern is identical in other European populations (Fig. 1f; Supplementary Fig. 8), and the biased enrichment remains when significance is assessed with a weighted block jackknife approach^21^, that accounts for LD in the tails (with the only exception of Toscani in Italy (TSI), Supplementary Fig. 9), and when we use only sites where the derived allele frequency is higher in Europe than in Africa (Supplementary Fig. 10). It also remains when we use Luhya (Luhya in Webuye, Kenya) rather than YRI although not always significantly so with the weighted block jackknife approach (Supplementary Fig. 11) probably due to the effect of recent Western Eurasian gene flow^25^. When we explore different genetic annotations (protein changing, conserved or regulatory) only putatively regulatory sites show a stronger enrichment in the European than the African tail, although the effect is due to the genic sites (Supplementary Note 1).

Our results thus indicate that genic alleles changed in frequency disproportionally in Europeans after divergence of the Ust′-Ishim population, a pattern absent in East Asians. Not surprisingly, simulations under a realistic demographic model of human populations^26^ (Fig. 2a; Supplementary Fig. 7) that include background selection but no positive selection cannot explain our observations (Fig. 1h; Supplementary Fig. 12). Simulations using a more complex demographic scenario in Europe, with several ancient groups including early hunter gatherers, farmers and Basal Eurasians (Fig. 2b; Supplementary Fig. 7 see below and Methods) also fail to explain the biased DAnc genic enrichment pattern (Supplementary Fig. 13). Allelic surfing (the random change in allele frequencies in the front of an expanding population^27^) is an unlikely explanation because it is not expected to be stronger in Europeans than in East Asians, as they both experienced similar population expansion^26^. Also, a specific transition that has higher mutation rate in Europeans than in other groups (codon TCC to codon TTC^28^) cannot explain the biased DAnc pattern because these alleles are overwhelmingly at low frequency^28^ and there is no overrepresentation of transitions in the DAnc European tail (rate of transitions: 0.39; resampling *P* value: 0.904). Finally, no allele in this tail is identified as having Neandertal origin in genome-wide maps of Neandertal introgression^29^.

Taken together, these results indicate that the biased European DAnc pattern is best explained by local adaptation in European populations. The absence of a similar signature in East Asians is likely due to the stronger drift experienced by these populations^24^, which increased the presence of neutral alleles in the East Asian tail. In any case, these results suggest that positive selection has played an important role in the (otherwise modest) differentiation of present-day human populations.

### DAnc with low-coverage ancient genomes and modern genomes

The benefit of using ancient genomes is illustrated in the fact that the DAnc analysis reveals a signature of local adaptation that is absent in similar approaches that use modern genomes alone (Fig. 1a,b). Yet, the Ust′-Ishim genome is of unusually high coverage and quality for an ancient sample, so we explored the effect of coverage on DAnc. Using data from a single Ust′-Ishim library with low coverage (1.6-fold) resulted in no significant genic enrichment in the European tail (Supplementary Fig. 14). This is due to both the erroneous inclusion and the erroneous exclusion of alleles in the tail (considering the 42-fold Ust′-Ishim DAnc tail our gold standard, the DAnc 1.6 × Ust′-Ishim tail shows a sensitivity of 0.6 and a specificity of 0.79). So when population samples are not available, high sequence coverage is necessary in this approach.

In addition, we explored the advantage provided by ancient genomes by replacing Ust′-Ishim in DAnc with one present-day human (we considered five high-coverage present-day humans of Asian or Oceanian ancestry from Meyer *et al*.^30^). These results (Supplementary Data 1) overall confirm the genic enrichment in the European tail. But results are inconsistent across the five genomes and the magnitude of the bias rarely reaches that obtained with Ust′-Ishim. This is likely due to the drift in this third, modern human population. Replacing Ust′-Ishim by allele frequency information from an East Asian population (Han Chinese South (CHS), CHB, or Japanese in Tokyo, Japan (JPT) from the 1000 Genomes), the number of alleles in the European tail is too low to perform meaningful analyses. Therefore using an ancient genome improves our power to infer the timing of allele frequency differentiation, a gain that will only grow with larger numbers of high-quality ancient genomes.

### The functional consequences of alleles in the genic tails

**Figure 3:**
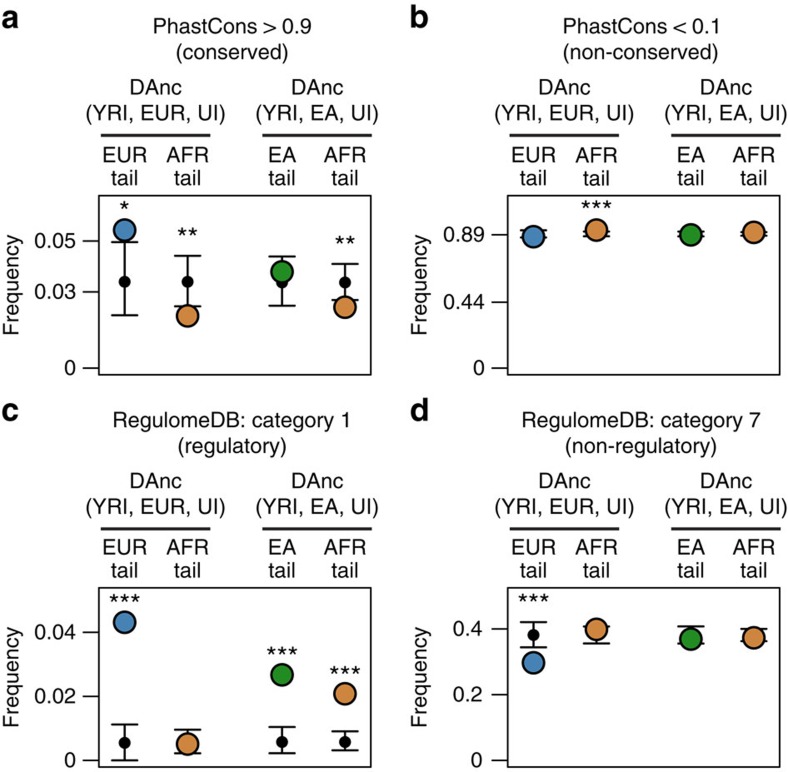
Conservation and putatively regulatory role of genic alleles in DAnc tails. Conservation was measured with phastCons^31^, with (**a**) phastCons >0.9 representing strongly conserved sites and (**b**) phastCons <0.1 non-conserved sites. Observed frequencies of genic alleles with different levels of phylogenetic conservation are shown for the DAnc(YRI, Europe, UI) tails and the DAnc(YRI, East Asia, UI) tails using a coloured dot (blue for European, green for East Asian, orange for African). On the basis of resampling from the entire distribution the mean expectation is shown as a black dot and the 95% confidence interval as black lines. Asterisks indicate significant differences between observations and expectation (*P<0.05, **P<0.01 and ***P<0.001). **c** and **d** show the same analysis for putative regulatory function according to regulomeDB^32^, with (**c**) regulomeDB category 1 representing likely regulatory sites and (**d**) category 7 representing sites with no evidence of a regulatory role. AFR, African (YRI); EA, East Asia; EUR, Europe; UI, Ust′-Ishim.

Natural selection targets only functional alleles, while random genetic drift affects sites regardless of functionality. Alleles with important functional consequences are thus expected to be largely constrained and unlikely to substantially change allele frequency solely due to drift. To further explore the contribution of drift and natural selection in the differentiation of genic alleles, we measured their evolutionary conservation and their putative functional effects. Phylogenetic conservation was assessed with phastCons^31^ on a 33-placental-mammal alignment excluding humans. The genic African tails are depleted of strongly conserved sites (Fig. 3a, *P* value <0.01), but not of non-conserved sites (Fig. 3b) when compared with equally sized sets of random SNPs. This is not surprising, as it reflects allele frequency changes during the out-of-Africa bottleneck affecting mostly weakly conserved sites. This pattern is absent in the European and East Asian tails (Fig. 3a,b), and it is actually reversed in the European tail, which is significantly enriched in strongly conserved sites (Fig. 3a, *P* value <0.05).

A similar comparison is not possible for non-synonymous and synonymous SNPs due to low numbers in the DAnc tails (5–23 non-synonymous SNPs), but is possible for sites with and without putative regulatory function according to regulomeDB (ENCODE)^32^. The genic European tail shows a significant enrichment in putatively regulatory sites (category 1, *P* value <0.001) and a significant depletion in sites with no evidence of regulatory function (category 7, *P* value <0.001; Fig. 3c,d). This suggests that the European tail is particularly enriched in functional sites, a result that is independent from the analyses above (only one tail allele is both in regulomeDB category 1 and phastCons >0.9). In the East Asian analysis, both tails show enrichment in regulomeDB category 1 SNPs (*P* value <0.001), but no depletion in category 7 SNPs (Fig. 3c,d).

The observed excess of strongly constrained and putatively regulatory alleles in the genic DAnc European tail is highly unlikely under a scenario of drift alone, but it is again compatible with the action of positive selection driving the changes in frequency of functionally relevant alleles.

### Highly differentiated alleles in ancient Europeans

Our understanding of European population history has improved considerably in recent years with the analysis of ancient DNA^33^,^34^,^35^. Current evidence suggests that during the Neolithic transition, early European farmers from the Near East colonized Europe and admixed with local hunter-gatherer populations^33^,^34^. So while the agricultural revolution largely replaced hunter-gatherer subsistence practices, Europeans display ancestry from both populations in their genomes. To understand the contribution of these two populations to the European DAnc tail, we analysed the high-quality genome of *Loschbour*, an ∼8,000-year old west European hunter gatherer, and of Stuttgart, an ∼7,000-year-old early European farmer^33^ that, like Ust′-Ishim, we consider representative of their respective populations (Fig. 2b). Importantly, these two genomes were produced by the same team and in parallel and their data are largely comparable^33^.

**Figure 4:**
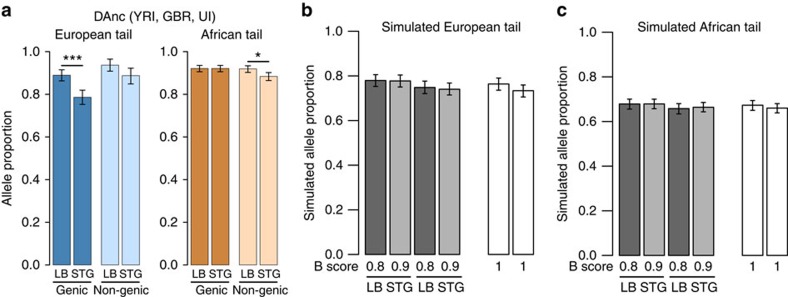
Analysis of the presence of European DAnc tail alleles in ancient European genomes. (**a**) Proportion of European alleles present in Loschbour (LB) or Stuttgart (STG) among those in the tails of the DAnc (YRI, GBR, Ust′-Ishim) distribution. Bootstrapping provided confidence intervals (95% confidence interval shown) and asterisks indicate significant differences between LB and STG (*P<0.05, ^**^P<0.01 and ^***^P<0.001). (**b**,**c**) Expectation of these proportions in the (**b**) European or (**c**) African tails, in the absence of adaptive evolution and with various intensities of background selection (measured by *B* scores). Genic alleles are simulated with *B*<1, and non-genic alleles with *B*=1. The 95% confidence interval shown is obtained via bootstrapping.

We explored the contribution of these two ancestral populations to the pool of highly differentiated European alleles. Specifically, we focus on the alleles in the tails of the European (GBR) DAnc distribution and ask whether the Loschbour and Stuttgart individuals carry the high-frequency European allele. In the genic European tail, Stuttgart has 10.4% fewer European alleles than Loschbour (Fig. 4a). This difference is significant (*P* value <0.001) and absent in non-genic alleles. As before, the signature is comparable among European populations (the increased proportion in Loschbour is 13.2% for Finnish from Finland (FIN), 7.6% for CEU, 7.6% for TSI; Supplementary Fig. 15) and the trend remains when we use only sites where the high-frequency allele in Europe is the derived one (Supplementary Fig. 16). The Stuttgart genome has a higher accumulation of transitions than the Loschbour genome (likely due to ancient DNA damage) but the pattern holds when we use only transversions (Supplementary Fig. 17) or homozygous sites (Supplementary Fig. 18). Therefore, our analysis shows that relative to Stuttgart, Loschbour carried more of the genic alleles that are today highly differentiated in European populations. Principal component analysis (PCA) analyses of these populations using SNPs in the different tails are largely consistent with this (Supplementary Fig. 19). This suggests that hunter gatherers contributed disproportionally to the highly differentiated alleles within genes in Europe but, intriguingly, not outside of genes.

Next, we tested whether this observation can be explained by demography and background selection alone. We used the established model of human demography^26^ with Ust′-Ishim^23^, and incorporated Loschbour and Stuttgart based on a previously inferred demographic model^33^ (Fig. 2b; Supplementary Fig. 7) and their observed heterozygosities (Supplementary Figs 20 and 21, Methods). We note that our model has a lower effective population size (Ne) in the hunter-gatherer population (Loschbour, Ne ∼400) than in the farmer population (Stuttgart, Ne ∼500), and it includes the basal Eurasian population, the proposed eastern non-African population that contributed genetically to the population of Stuttgart (Fig. 2b)^33^. Figures 4b,c show the results of our simulations under increasing levels of background selection. The number of highly differentiated European alleles in Stuttgart is lower than in Loschbour (due to the lower Ne of Loschbour in our model), but the difference is not significant and is smaller (on average 3.4% less) than in the actual genomes, regardless of the strength of background selection simulated (Supplementary Fig. 22). Additional models with very extreme (even unrealistic) values of Ne in either population also failed to reproduce our observations in magnitude and significance of the enrichment (Supplementary Fig. 23). While the Ne of these populations is uncertain, this analysis shows that even strong variation in Ne does not account for our observations. Thus, neutrality and background selection do not explain the patterns observed.

Available genetic information for other ancient European individuals^15^,^17^,^18^,^34^,^35^,^36^,^37^,^38^ could unfortunately not be used. This is because these individuals are either younger than Loschbour and Stuttgart (and thus less informative in our approach), have low average sequence coverage of their genomes (hampering the use of genome-wide individual genotypes) or do not represent well-defined populations in time and space (hindering our ability to accurately estimate past allele frequencies). An important exception is NE1, the genome of a 7,000-year old farmer from Hungary, with an average genome coverage of ∼22 × (ref. 16). Interestingly, NE1 and Stuttgart are equally close to present-day European populations, but NE1 is slightly closer (not significantly) to the hunter gatherer than Stuttgart is (based on D-statistics, *Z* score=1.93, Supplementary Table 1 and PCA, Supplementary Fig. 24; see also Supplementary Note 2). This is consistent with gene flow between the populations of Loschbour and NE1. Like Stuttgart, NE1 has fewer alleles from the DAnc genic European tail than Loschbour (Supplementary Fig. 25), although the difference between the two genomes is not significant, consistent with Loschbour-to-NE1 gene flow.

We conclude that the genomes of hunter gatherers contained many putatively advantageous alleles that are found at very high frequency in present-day Europeans, and that are absent from the genome of a contemporaneous farmer (Stuttgart). This is consistent with hunter gatherers, who lived in Europe for thousands of years before the later arrival of farmers, having already adapted to their local European environments. Interestingly, the genome of a farmer that shows modest evidence of admixture with hunter gatherers (NE1) bears already some of these alleles.

### Biological function of SNPs in the European tail

**Figure 5:**
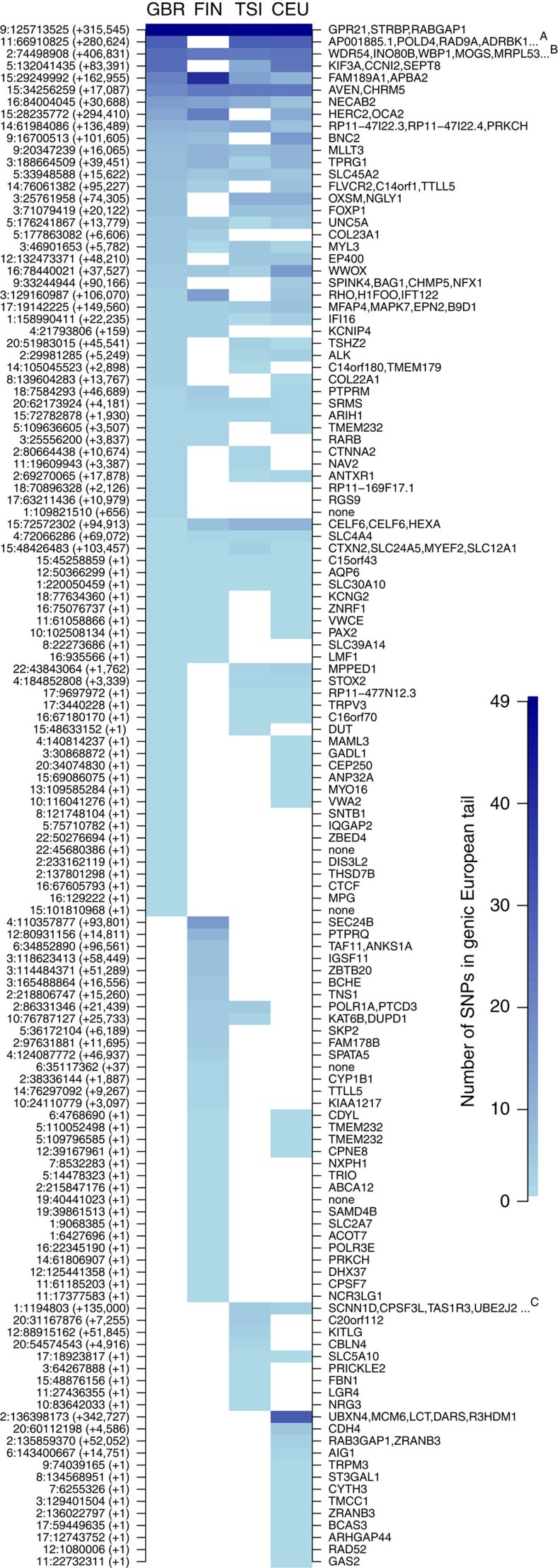
Genic alleles in the DAnc(YRI, Europe, UI) tail and overlapping genes. Close-by alleles are combined in a region if they are <100-kb apart. The left side of the figure shows the chromosomal position (chr:start) and the length in bp of each region. The right side shows the genes that overlap with each region. The number of alleles in each genic DAnc tail (*P*_2_: GBR, FIN, TSI, CEU) is shown with the heatmap. Columns are ordered by number of alleles starting left. Superscripts note regions with too many genes to fit the figure: ^A^: ANKRD13D, SSH3, CLCF1, PPP1CA, TBC1D10C, CARNS1, KDM2A; ^B^: CCDC142, PCGF1, DQX1, AUP1, DCTN1, C2orf81, RTKN, LBX2, TLX2, HTRA2, LOXL3, DOK1, SEMA4F, TTC31, M1AP, RP11-287D1.3, SLC4A5; ^C^: ACAP3, PUSL1, GLTPD1, DVL1, MXRA8, CCNL2, AURKAIP1.

The alleles in the genic European DAnc tail are good candidates to have contributed to local adaptation, although obviously this tail likely contains some neutral alleles too. All SNPs in this tail are presented in Fig. 5 and Supplementary Data 2. We further explored whether sets of them are enriched in certain gene ontology (GO) categories. This analysis benefits from larger numbers of SNPs, so we considered the union of all alleles in the genic European tail for the DAnc analysis between the African YRI and any European (GBR, FIN, TSI, CEU) population. We also divided this set into those where the high-frequency European allele is exclusively present either in Loschbour or Stuttgart. Each SNP set was turned into a gene set and GO enrichment analyses were run using 100,000 random SNPs as background (Methods); each gene is included only once even if it contains several SNPs in the set (that is, linked SNPs do not affect the signature).

**Figure 6:**
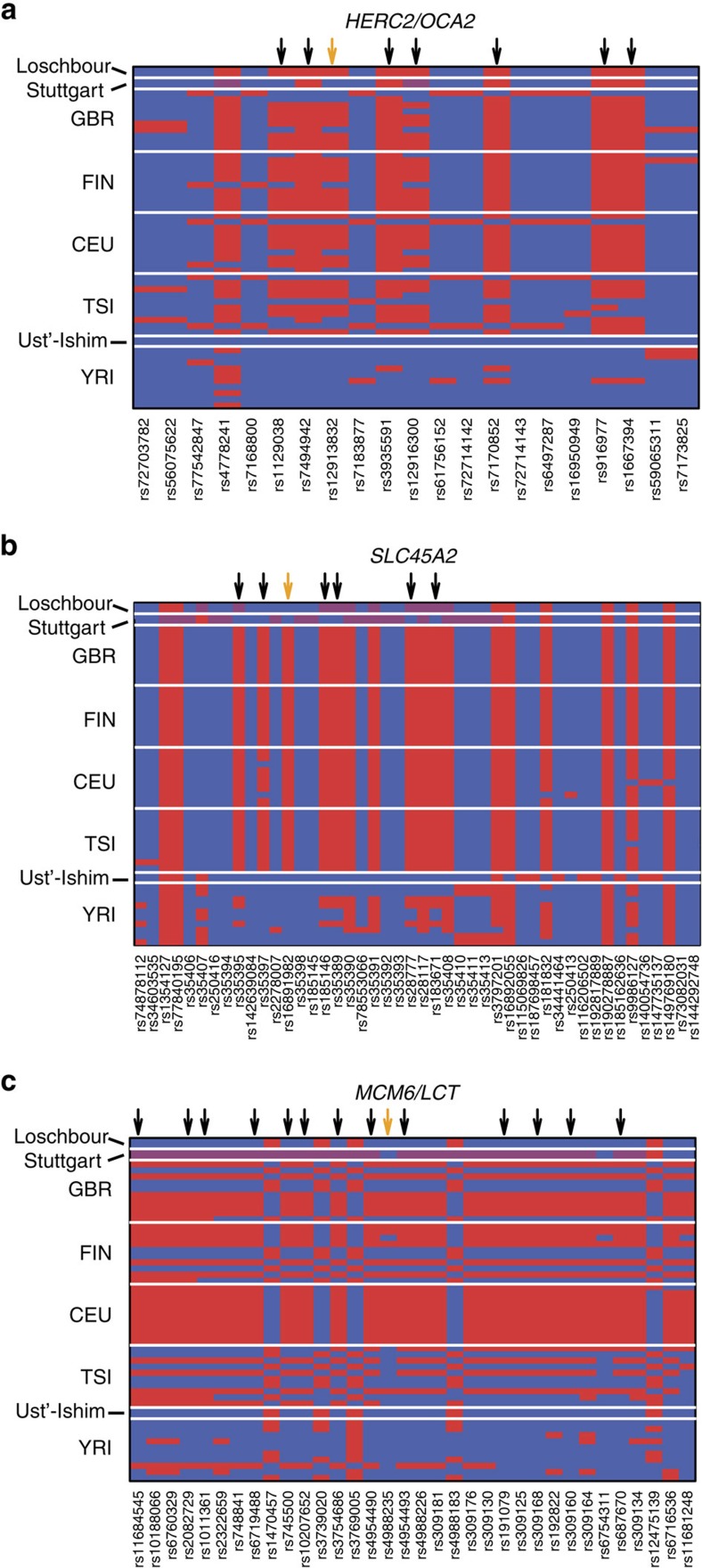
Haplotypes in ancient and present-day modern humans. Haplotypes for (**a**) *OCA2/HERC2*, (**b**) *SLC45A2* and (**c**) *LCT/MCM6*. SNPs were included if they show moderate to strong LD with the phenotypically relevant SNP (*OCA2/HERC2*: rs12913832, *SLC45A2*: rs16891982, *LCT/MCM6*: rs4988235), which is marked with an orange arrow. Black arrows indicate alleles that are present in at least one European DAnc tail (*P*_2_: GBR, FIN, TSI or CEU). The heatmap shows the phased haplotype for the 1000 Genomes data set (red derived allele and blue ancestral allele) and the genotype for the ancient genomes (red homozygous derived, purple heterozygous and blue homozygous ancestral).

We identify one single significantly enriched functional category (cellular component): melanosome membrane (*P* value=0.004, Supplementary Table 2) for the European tail alleles present exclusively in Loschbour. Two genes drive this signature: *OCA2* with 10 SNPs and *SLC45A2* with 5 SNPs. Both of them harbour variation known to affect pigmentation^39^ and have classical signatures of positive selection^40^,^41^, recently supported by a targeted SNP analysis in several ancient European samples^17^,^19^. The derived allele upstream of *OCA2* (rs12913832 in *HERC2*) is associated with blue iris colour in Europeans^42^ and light skin pigmentation^43^; both this variant and its linked variation show that Loschbour carried the predominant European haplotype (Fig. 6a). The derived allele in *SLC45A2* non-synonymous rs16891982 is associated with lighter skin pigmentation and increased melanoma risk in Europeans^44^,^45^. No ancestral genome carries rs16891982's derived allele, but Loschbour carries the haplotype that, in present-day populations, is linked to the derived allele (Fig. 6b). Therefore hunter-gatherer populations likely contributed both *OCA2* and *SLC45A2* advantageous alleles to the European gene pool. This agrees well with these populations inhabiting northern European areas before the arrival of southern farmer groups. Lighter skin pigmentation has been proposed to be advantageous in northern latitudes to sustain vitamin D_3_ production in low-ultraviolet environments^46^. Blue iris colour may be advantageous to reduce the risk of seasonal affective disorder (also known as winter depression) in high latitudes^47^, increase sensitivity to glare^48^ or for sexual selection^49^.

Another interesting target of positive selection in Europe is lactase persistence (LP)^5^,^50^. It has been proposed that LP was introduced in Europe during the Neolithic transition and the introduction of farming culture^51^,^52^. It is known that the two derived alleles associated with LP in Europe (in rs4988235 and rs182549)^53^ are absent in the two ancient genomes^33^ and are not observed in Europe until ∼2300 BC in an individual of inferred steppe ancestry^17^. But the European tail includes a large number of alleles in the lactase enhancer region and the LP haplotype (chr2:135859371-136740900) that are exclusively present in Stuttgart (65% of Stuttgart specific targets; Fig. 6c). Thus the haplotype that is today associated with LP in Europe originated most likely in this genetic background, which we detect only in the Stuttgart farmer, although this individual itself did not carry the LP allele. *SLC45A2* and *Lactase* exemplify the advantage of using genomic information and haplotypes to identify the source population of interesting alleles, as approaches based solely on the associated SNPs (ignoring linked variation) depend fully on the chance that the sequenced individuals happen to carry it.

### The information in ancient genomes

We present a joint analysis of present-day and ancient genomes to study genome-wide, worldwide patterns of modern human population differentiation. The accuracy of the DAnc statistic and our approach is limited by the availability of high-quality ancient genomes of old ages (here Ust′-Ishim^23^), but there is little doubt that the advent of larger numbers of ancient genomes will significantly improve our knowledge not only of demographic history, but also of the genetic adaptation of human (and non-human) populations to their local environments. In fact, studies based on pre-selected interesting SNPs in ancient European populations^15^,^16^,^17^,^18^,^19^ are already contributing to the identification of selective sweeps.

In this case, high-quality ancient genomes allow us to globally isolate the effect of the out-of-Africa bottleneck, providing valuable information about the genetic differentiation that accompanied the colonization of Eurasia. This shows that only 30% of the most strongly differentiated alleles between Africans and Eurasians rose in frequency after the out-of-Africa bottleneck, during the colonization of Eurasia. Nevertheless, our results provide clear evidence that local adaptation contributed to these allele frequency changes in European populations, as strongly differentiated alleles in Europeans are enriched in likely functional variants: genic, previously constrained and putatively regulatory. Importantly, only the use of ancient genomes allows us to show that the pattern cannot be explained by background selection or demography alone. Interestingly, European hunter gatherers contributed disproportionally to these putatively selected alleles, which are enriched in genes associated with variation in pigmentation. This agrees well with hunter gatherers having adapted to northern European environments before the arrival of southern farmers, and it suggests that these adaptations soon became important for the admixed populations too.

## Methods

### Present-day modern human genomes

We analysed genome-wide data from the 1000 Genomes (phase I release)^20^. We considered (1) autosomal variants detected in the low-coverage sequencing to ensure similar data quality across the genome, and (2) African and Eurasian populations with information for at least 50 unrelated individuals, which was met by 10 populations in three continents (African ancestry: YRI, Luhya in Webuye, Kenya); European ancestry: GBR, CEU (Utah residents (CEPH) with Northern and Western European ancestry), FIN, TSI; East Asian ancestry: CHS, CHB, JPT). African-Americans (African Ancestry in Southwest US) were excluded because of recent European admixture^20^. To define derived allele frequency, we used the ancestral allelic state in the 1000 Genomes data (based on the Ensembl 59 comparative 32 species alignment^54^) using only SNPs with a high-confidence ancestral inference and excluding indels due to their cryptic variation patterns^55^. Phase III 1000 Genomes data were not used as the high-coverage exome data and the low-coverage whole-genome data are jointly used for SNP calling, likely creating differences in the quality of the genotypes between genic and non-genic regions. To keep all analyses comparable, we considered for all analyses only sites where there is genotype for Ust′-Ishim too (see below).

In addition, we also analysed high-coverage genomes from Meyer *et al*.^30^, which are comparable in quality to the ancient genomes we analysed. From the 11 genomes available, we analysed individuals with the following ancestry: Dai (SS6004467, Asia); Han (SS6004469, Asia); Papuan (SS6004472, Oceania) and two Australian (SS6004477, Oceania and SS6004478, Oceania). They provided reasonable outgroups, and thus sensible replacements for the ancient Ust′-Ishim genome (*A*), in the DAnc analysis with Africans and Europeans as *P*_1_ and *P*_2_, thus we focused on these five genomes. All genomes were processed and filtered following Meyer *et al*.^30^ according to: (1) mapability (35mers aligning to one single location in the genome, allowing for one mismatch), (2) map quality of reads covering a position (MQ≥30), (3) upper and lower coverage (eliminating the 2.5% of positions with the highest or the lowest coverage from each sample) and (4) simple repeats (filtering out regions present in hg19 tandem repeat finder track at UCSC (http://hgdownload.soe.ucsc.edu/golden Path/hg19/database/simpleRepeat.txt.gz)). To ensure full reciprocal overlap of sites across individuals, we excluded positions that did not pass all filters in all individuals considered in each particular analysis.

### Ancient modern human genomes

Ancient genomic data was processed as described in the original publications of Ust′-Ishim^23^, and Loschbour and Stuttgart^33^. An identical set of filters was applied as to the modern present-day genomes above.

We also explored a low-coverage genomic data set of Ust′-Ishim to assess the effects of coverage in the DAnc analysis. We used the library B5347 (ref. 23), which yielded a genome-wide coverage of 1.6x. Since genotype calling is not possible, we inferred a genotype (0 or 1) for each position by a randomly sampling one read. We then filtered this data like the high-coverage Ust′-Ishim data (see above). Considering the high-coverage genotypes as the gold standard we assessed sensitivity and specificity of the low-coverage Ust′-Ishim DAnc results in the DAnc genic European tail using *P*_1_: YRI and *P*_2_: GBR.

### Annotations

To explore population differentiation in the light of genomic annotation the data were divided into genic and non-genic positions. We used the Ensembl Gene annotation (GRCh37.p13) start and end coordinates of all autosomal, protein-coding transcripts. Genic regions were defined as the transcript plus 2 kb at the start and the end of those regions. All remaining regions were defined as non-genic.

### Enrichment analysis with present-day populations

Following Coop *et al*.^2^, we compute the enrichment in genic alleles versus all alleles (genic plus non-genic SNPs) across the distribution of allele frequency differences in a pair of populations. Allele frequency differentiation was calculated subtracting population A from population B and the genome-wide distribution of allele frequency differences was binned in bins of size 0.2. For each bin we calculate the ratio of genic over genic plus non-genic alleles and normalize by the genome-wide ratio of genic over all alleles. This provides a measure of enrichment (or depletion) of genic alleles for each bin.

CIs were calculated based on two different approaches: (1) bootstrap (re-sampling with replacement) of all SNPs in the genome and full genic enrichment analysis; 10,000 iterations provide a bootstrap distribution of genic enrichment in each allele frequency differentiation bin. (2) Weighted block jackknife^56^,^57^,^58^ following Busing *et al*.^21^ and using 200-kb genomic blocks (the block size in ref. 2), the block jackknife approach accounts for LD among sites in each allele frequency differentiation bin (particularly in the tails, as LD is an outcome of recent selective sweeps); the weighted approach down-weights, within each bin, the blocks with larger numbers of SNPs as they are presumably in LD and contribute non-independent signatures.

The CIs were used to test for a significant enrichment in genic alleles (that is, whether the ratio of genic versus genic plus non-genic significantly departs from genome-wide expectations, here 1). The overlap between the CI of both tails was then used to test for a bias in the genic enrichment (that is, if the enrichment is significantly larger in one tail compared with the other). We obtained the 95%, 99% and 99.9% CI, which in the two tests above translated into significance levels of *, ** and ***, respectively.

### DAnc statistic

Each DAnc analysis was performed following formula (2) in the main text. We considered only positions with genotype information in all three populations (*P*_1_, *P*_2_ and *A*), and that are polymorphic between both present-day populations (*P*_1_, *P*_2_). Following the two-population analysis above, we divide the genome-wide distribution of DAnc scores in bins of size 0.2, calculate the ratio of genic alleles over all alleles (genic plus non-genic) in each bin and normalize by the genome-wide ratio. This provides a measure of enrichment of genic alleles for each DAnc bin. CIs were calculated as above (bootstrap, weighted block jackknife) and used to test for a genic enrichment and for a biased enrichment between the two tails.

### Simulations

For the present-day humans, we performed coalescent simulations without positive selection to explore the effects of demography and background selection in our analysis. We performed 400,000 neutral simulations using ms^59^ for a 2-kb locus (to avoid effects of recombination). Simulations are based on the population-genetic model proposed by Gravel *et al*.^26^, with modifications following the models proposed in Fu *et al*.^23^ and Lazaridis *et al*.^33^ to include Ust′-Ishim and the ancient Europeans, respectively, in that model (details below). We consider a generation time of 25 years and a mutation rate of 2.36 × 10^−8^ per site per generation, as these were the parameters used to infer the demographic model^60^. Following Coop *et al*.^2^, the effect of background selection can be approximated as reduced local effective population size (which increases drift). We thus model background selection as a reduction in the effective population size in our simulations, by a factor that represents the so-called *B* score^61^. We explore a range of *B* scores and present here results for 0.8 and 0.9 (where Ne is multiplied by 0.8 and 0.9, respectively), which represent realistic values for the human genome^61^ (Supplementary Fig. 26) and resemble best of our observations (*B* scores <0.8 resulted in extremely high levels of genic enrichment in both tails and did not affect the bias between the two tails). Simulations under a *B* score of 1 (that is, no background selection) are used as our proxy for non-genic in real data, and lower *B* scores are used as proxy for genic in real data. Our enrichment statistic is thus the ratio between sites from simulations with *B*=1 and simulations with a lower *B* score (0.8 or 0.9). Binning and genic enrichment were performed as in real data.

For Ust′-Ishim, we included in our demographic model the 45,000 years old ancient Ust′-Ishim individual as proposed in Fu *et al*.^23^ (SOM pg. 111). We visualized the demographic model in detail in Supplementary Fig. 7. Here, the Ust′-Ishim population branches off 2,000 generations ago from the population that experienced the out-of-Africa event, and it keeps a constant effective population size (Ne) of 1,860 until it is sampled 1,800 generations before present (∼45,000 years ago) (Fig. 1g,h). As in Fu *et al*.,^23^ we simulate branch shortening by splitting at the time of sampling the Ust′-Ishim population into two populations with very small Ne, and removing all mutational differences between them (which removes all mutations since the time of sampling). In addition, we explored the putative effects of variation in the Ne of the Ust′-Ishim population (Ne=186 or Ne=18,600) (Supplementary Fig. 12), although changes in Ust′-Ishim Ne are not expected to affect our results.

For the ancient Europeans, we incorporated the ancient European samples into our model using the three-population mixture demographic model presented in Lazaridis *et al*.^33^ with a focus on the branches directly affecting Loschbour and Stuttgart samples (Fig. 2b). We visualized the demographic model in detail in Supplementary Fig. 7. Here, this model involves the following modifications (forward in time):

- Basal Eurasians branch off the out-of-Africa population 2,300 generations ago with an Ne=1,860 (same as the out-of-Africa population Ne). The Basal Eurasian population is a hypothetical ancient Eastern non-African population that is believed to have contributed to the Stuttgart farmer but not to the Loschbour hunter gatherer^33^.
- Immediately after the Eurasian split (2,000 generations ago) the European branch splits into two populations representing farmers and hunter gatherers.
- To account for possible differences in drift between the ancient European populations we allowed for independent population-specific changes in the Ne of both the hunter gatherer and the farmer populations for 100 generations (∼1,900–2,000 generations ago; details below).
- Both the hunter-gatherer and the farmer populations experience growth (∼1,900–present) following the European growth in Gravel *et al*. 26
- The Basal Eurasian population merges with the farmer population (∼1,900 generations ago) at a ratio of 56:44 (ref. 33).
- Loschbour is sampled from the hunter-gatherer population 320 generations ago (∼8,000 years ago).
- Stuttgart is sampled from the farming population 280 generations ago (∼7,000 years ago).
- The farmer and the hunter-gatherer populations merge in equal proportions 280 generations ago.

Variation in drift between Loschbour and Stuttgart might affect the contribution of highly differentiated genic alleles by one of the two ancient Europeans. The demographic history of these populations is not known, so we account for putative changes in Ne by including an independent Ne change for 100 generations in the history of each population. We infer these Ne based on the observed heterozygosity in the two ancient European samples. We first fitted the heterozygosity measured in Loschbour (*mlrho*) in Lazaridis *et al*.^33^ to neutral simulations using our demographic model. For each Ne, ranging from 50 to 5,000 in steps of 50, we ran 100,000 simulations and calculated the expected heterozygosity (Supplementary Fig. 20). For Loschbour, the measured heterozygosity is 4.75, which is best fit by a simulated Ne of 400 (Supplementary Fig. 20). The heterozygosity of Stuttgart exceeds the observed heterozygosity of other non-African populations, an unexpected pattern likely driven by an excess of transitions due to residual ancient DNA deamination^33^. Transversions are less affected by DNA damage and thus are more reliable in Stuttgart, so we inferred Stuttgart's Ne based on transversions only. Specifically, and given that the ratio of transversions between two samples reflects the ratio of their heterozygosities (*H*), we calculate Stuttgart's population Ne based on the inferred Loschbour's population Ne:


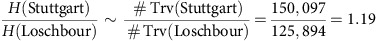


This inference leads to an Ne estimate of 483 for Stuttgart (Supplementary Fig. 21). There is one additional population, the Basal Eurasians that is inferred to have contributed genetically to Stuttgart and for which we have no genetic information^33^. We thus vary the Ne of the Basal Eurasians by an order of magnitude (Ne=186 or Ne=18,600), although this has a weak effect on the proportion of DAnc European tail alleles present in Stuttgart when compared with Loschbour (Supplementary Fig. 21). While it is clear that these Ne estimates may not be realistic, this exercise shows that reasonable variations in the Ne parameters do not affect, qualitatively, our results. To further explore the effect of more extreme differences in drift we ran our model with different combinations of Ne in Loschbour and Stuttgart (500, 1,032, 10,320) and in the Basal Eurasian population (Ne of 500 in addition to 1,860) (Supplementary Fig. 23). We obtained the 95% CI for each simulations by resampling 1,000 times the number of alleles observed in the European (*N*=625) or African (*N*=1,357) DAnc tail from the complete, respective simulation distribution.

### Inference of Neanderthal introgression

We explored if Neanderthal introgression into Eurasian populations^57^ contributes to the genic European DAnc tail, although regions of Neanderthal ancestry rarely reach allele frequency of 0.8 or higher in present-day Europeans^29^. We used the map of introgressed haplotypes in Eurasian individuals from Vernot *et al*.^29^. The other available introgression map^62^ was not used because its posterior introgression probabilities increase with the frequency of the derived alleles. We considered an allele as putatively introgressed if it falls in a region identified as being of Neanderthal ancestry^29^, and the derived allele has a frequency lower than 5% in YRI (Africa).

### Analysis of conservation and predicted regulatory effects

To assess conservation in the tails of the DAnc distribution, we used the phastCons program to estimate genome-wide conservation scores^31^. The conservation score for each site is based on a multiple, whole-genome alignment of 33 placental mammals (excluding humans) downloaded from UCSC, which is used to predict conserved elements. PhastCon scores range from 0 to 1 and we considered sites with conservation scores >0.9 as strongly conserved, and sites with conservation scores <0.1 as not conserved.

Predicted regulatory effects of sites were obtained from regulomeDB^32^ and downloaded from http://www.regulomedb.org/downloads. RegulomeDB is a public database that contains annotation of human genetic variants with known and predicted regulatory elements, (for example, regions of DNAase hypersensitivity, binding sites of transcription factors, or promoter regions), according to public data sets from GEO^63^, the ENCODE project^64^ and published literature. RegulomeDB scores range from 1 to 7. Category 1 contains sites with evidence at least for an eQTL and transcription factor binding or DNase peak, and are thus interpreted as regulatory important. Sites in category 7 do not show any evidence of regulatory effects. DAnc enrichment plots for these categories are presented in Supplementary Figures 27-31.

In addition, we explored if any European, East Asian or African tail show excess or depletion of phylogenetically conserved or putatively regulatory sites. We investigated the genic and non-genic DAnc tails using YRI as *P*_1_ and as *P*_2_ either Europeans (GBR, FIN, TSI or CEU) or East Asians (CHB, CHS or JPT). For each tail we compared the proportion of strongly conserved/not conserved and regulatory/not regulatory alleles in real data with its random expectations, which we obtained by resampling 5,000 times random alleles from the corresponding DAnc distribution to fit the number of alleles in the tail.

### Gene ontology analysis

Gene ontology analysis was performed using the software package FUNC^65^. We analysed the union of all alleles in the four European DAnc tails (with *P*_1_=YRI and *P*_2_=GBR, FIN, CEU or TSI), for SNPs that fall into protein-coding genes (Ensembl Gene annotation (GRCh37.p13)). We created two additional subsets of SNPs, according to whether the derived allele of the SNP in the DAnc European tail was exclusively present in Loschbour or exclusively present in Stuttgart. As background we used a random sample of 100,000 SNPs from all remaining genic SNPs. This approach corrects for spurious results due to long genes and/or variation in mutation rates. The categories reported in the main text were also observed when all SNPs absent in the DAnc tails were used as background; in these analyses a few additional categories appeared significantly enriched (Supplementary Table 2) but we do not discuss them since they were not observed in the smaller (more stringent) background. SNPs were assigned to genes if falling between the start and end location of the respective gene and each gene was considered only once for analysis (regardless of the number of SNPs in a certain set assigned to it).

### Haplotype analysis

Haplotypes were plotted to visualize specific target regions across samples. Variants were chosen from a 10-Mb region surrounding the focal SNP of each candidate gene (*HERC2*/*OCA2*: rs12913832; *SLC2A45*: rs16891982; *MCM6*/*LCT*: rs4988235). We consider only variants polymorphic in YRI or any European sample and that had data in all three ancient genomes. To simplify the plot we used only variants in high to moderate LD with the focal SNP (*OCA2*: *r*^2^>0.13; *SLC45A2*: *r*^2^>0.48; *LCT*: *r*^2^>0.6) in GBR for *OCA2* and *LCT*, and in FIN for *SLC45A2* (because the focal SNP in *SLC45A2* is fixed in GBR) calculated with vcftools^66^. Genotypes were plotted for the ancient individuals and 10 phased haplotypes for random YRI and European samples using the R package *adegenet* v.1.4-2 (ref. 67).

### Available computer code

The shell and R code used for all major analyses is available on our website as a tarball file (https://bioinf.eva.mpg.de/download/PopDiff_aDNA_study/) and in Supplementary Data 3 as a zip file. The archive files also contain a readme file documenting the application of the code.

## Additional information

**How to cite this article:** Key, F. M. *et al*. Human adaptation and population differentiation in the light of ancient genomes. *Nat. Commun.* 7:10775 doi: 10.1038/ncomms10775 (2016).

## Supplementary Material

Supplementary Figures, Supplementary Tables, Supplementary Notes and Supplementary References Supplementary Figures 1-31, Supplementary Tables 1-2, Supplementary Notes 1-2 and Supplementary References

Supplementary Data 1 DAnc results using present-day modern humans instead of Ust'-Ishim. DAnc analysis was performed for YRI as P1 and the European or East Asian populations as P2, and the ancient Ust'-Ishim genome was replaced with a single high-coverage genome from the *B team* presented in Meyer et al.^11^. We used all individuals of Asian or Oceanian ancestry (SS6004467...Dai, Asia; SS6004469...Han, Asia; SS6004472...Papuan, Oceania; SS6004477 and SS6004478, Australia, Oceania). The enrichment, the number of alleles in each tail, and the different confidence intervals are shown for (A) based on bootstrap or (B) based on a weighted block jackknife with 200kb genomic blocks; the significance of the bias between the two tails is indicated by asterisks (* < 0.05, ** < 0.01, and *** < 0.001).

Supplementary Data 2 List of genic alleles in the DAnc European tail. Union of alleles in the tails of all European DAnc (YRI, P_2_, Ust'-Ishim) distribution (P_2_: GBR, FIN, TSI, or CEU) including the genomic position (hg19), rs ID (dbSNP b138), and gene overlapping the SNP position. Table published alongside the manuscript as a tsv file.

Supplementary Data 3 Zip archive containing the shell and R code used for all major analyses. A tarball is also available on our website (https://bioinf.eva.mpg.de/download/PopDiff_aDNA_study/).
