# Supplementary material for: Human adaptation and population differentiation in the light of ancient genomes
Source: Nat Commun. 2016 Mar 18;7:10775. doi: 10.1038/ncomms10775 (PMC4802047; doi:10.1038/ncomms10775)
Supplement: Supplementary Figures, Supplementary Tables, Supplementary Notes and Supplementary References — Supplementary Figures 1-31, Supplementary Tables 1-2, Supplementary Notes 1-2 and Supplementary References [file ncomms10775-s1.pdf]

Supplementary Figures

A

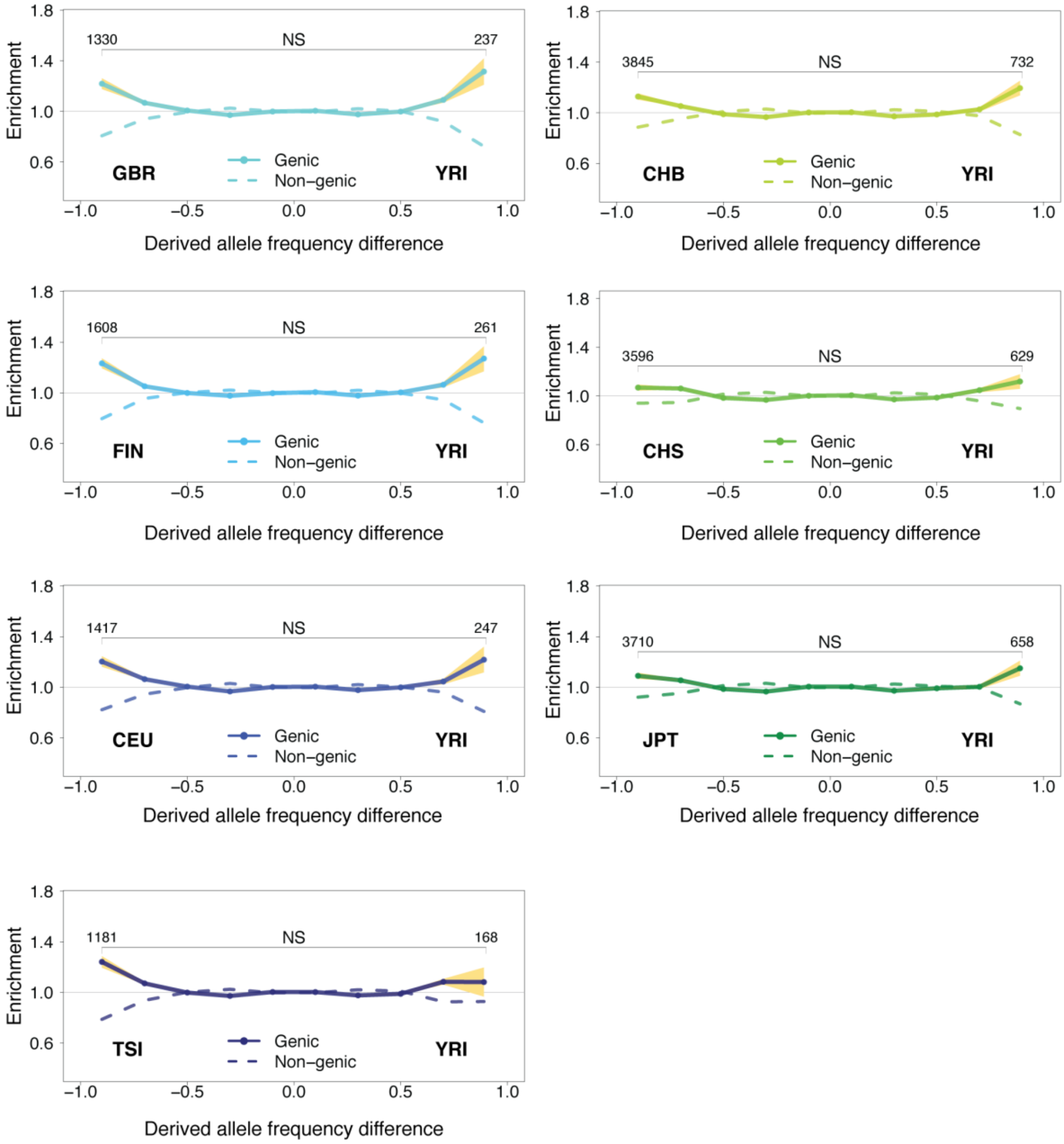

B

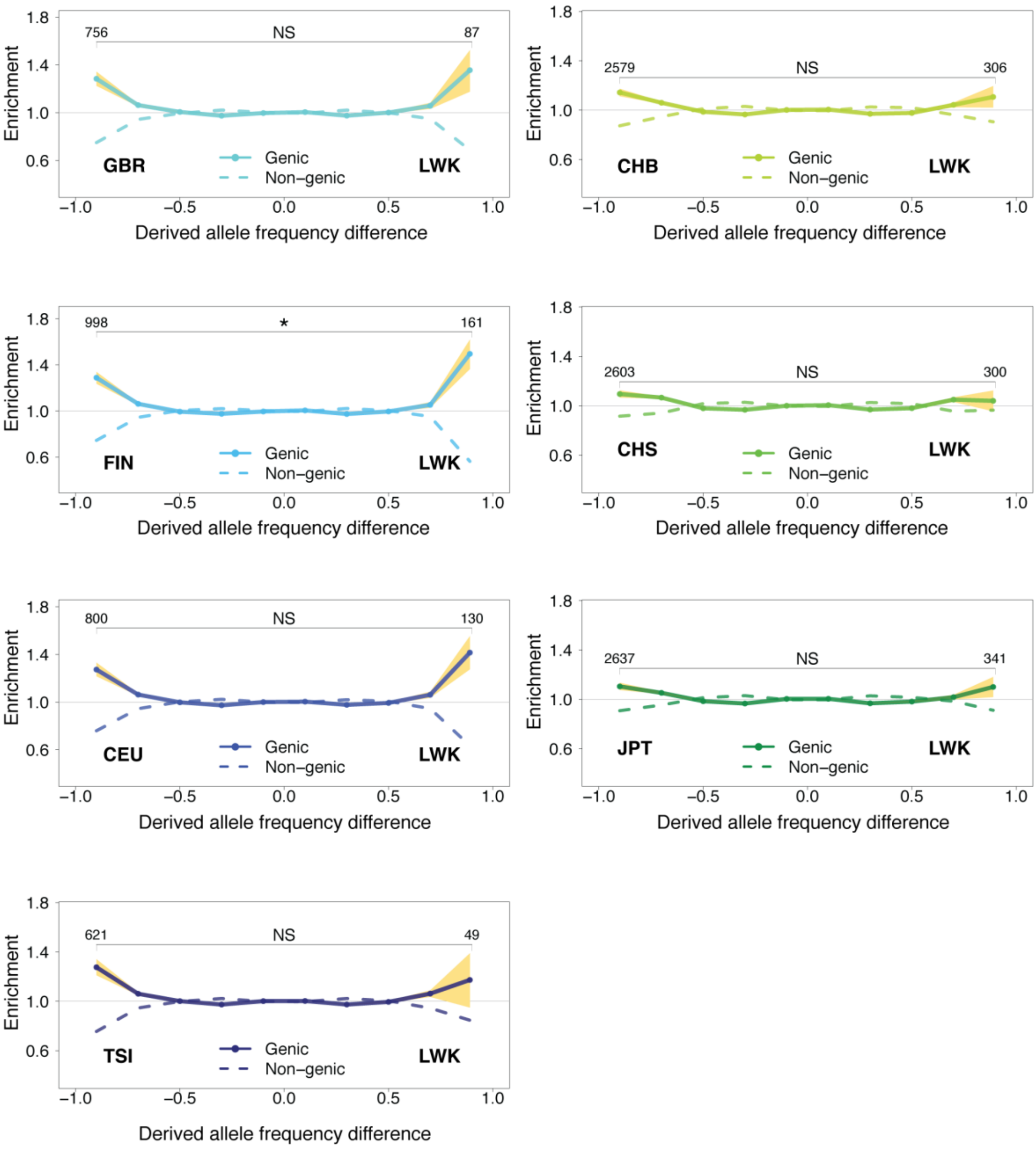

Supplementary Figure 1. Analyses of present-day population differentiation. (**A, B**) Enrichment of strongly differentiated genic alleles for all present-day population comparisons using all European and East Asian populations versus (**A**) YRI or (**B**) LWK. The bootstrap 95% confidence interval is shown in yellow, and the level of significance of the bias in genic enrichment when comparing the two tails is shown on top (* < 0.05, ** < 0.01, and *** < 0.001, and NS for non-significant). The number of genic SNPs in each tail is also shown.

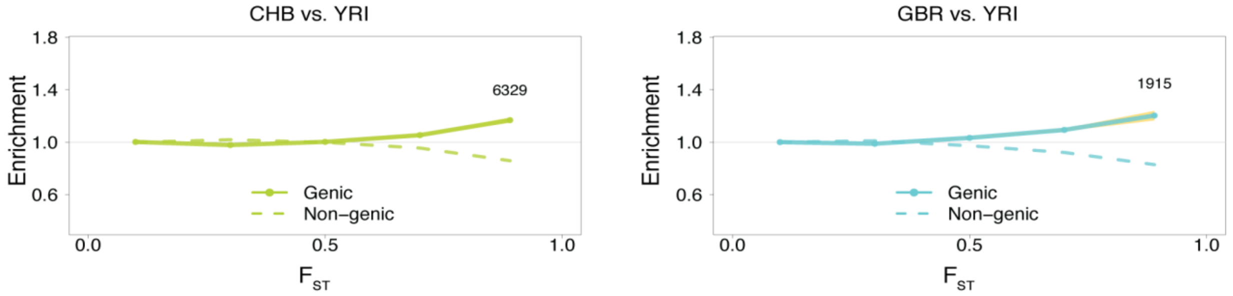

Supplementary Figure 2. Analyses of present-day population differentiation using F_{ST}. F_{ST}[1], a measure of population differentiation, was calculated for all SNPs variable in the comparison between (**A**) CHB and YRI or (**B**) GBR and YRI. The genic enrichment was calculated as in Figure 1. The number of sites in the far tail of the F_{ST} distribution (>0.8) is shown on top, and the bootstrap 95% confidence interval is shown in yellow.

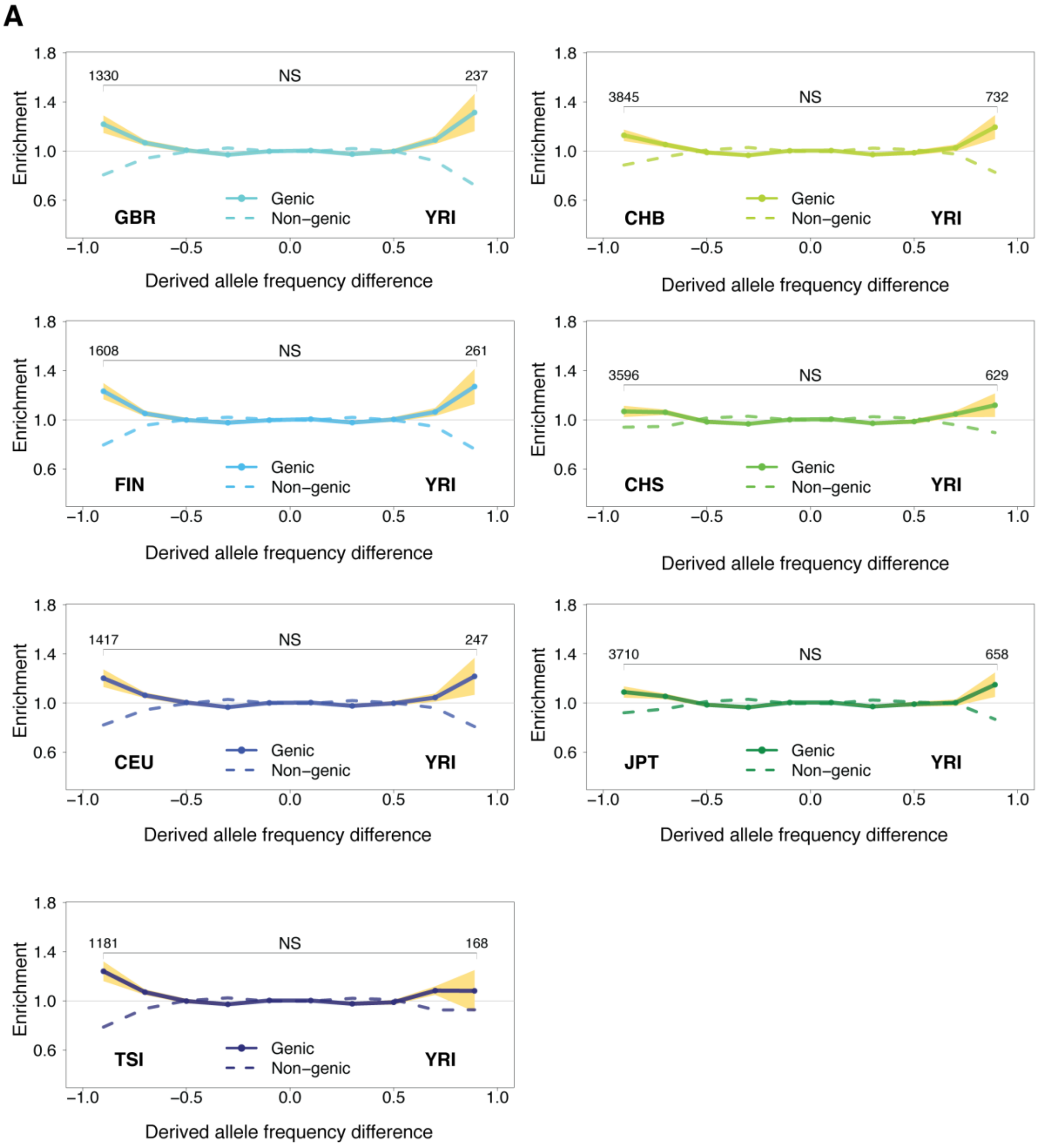
A
1330
NS
237
Enrichment
GBR
Genic
Non−genic
YRI
Derived allele frequency difference
3845
NS
732
Enrichment
CHB
Genic
Non−genic
YRI
Derived allele frequency difference
1608
NS
261
Enrichment
FIN
Genic
Non−genic
YRI
Derived allele frequency difference
3596
NS
629
Enrichment
CHS
Genic
Non−genic
YRI
Derived allele frequency difference
1417
NS
247
Enrichment
CEU
Genic
Non−genic
YRI
Derived allele frequency difference
3710
NS
658
Enrichment
JPT
Genic
Non−genic
YRI
Derived allele frequency difference
1181
NS
168
Enrichment
TSI
Genic
Non−genic
YRI
Derived allele frequency difference

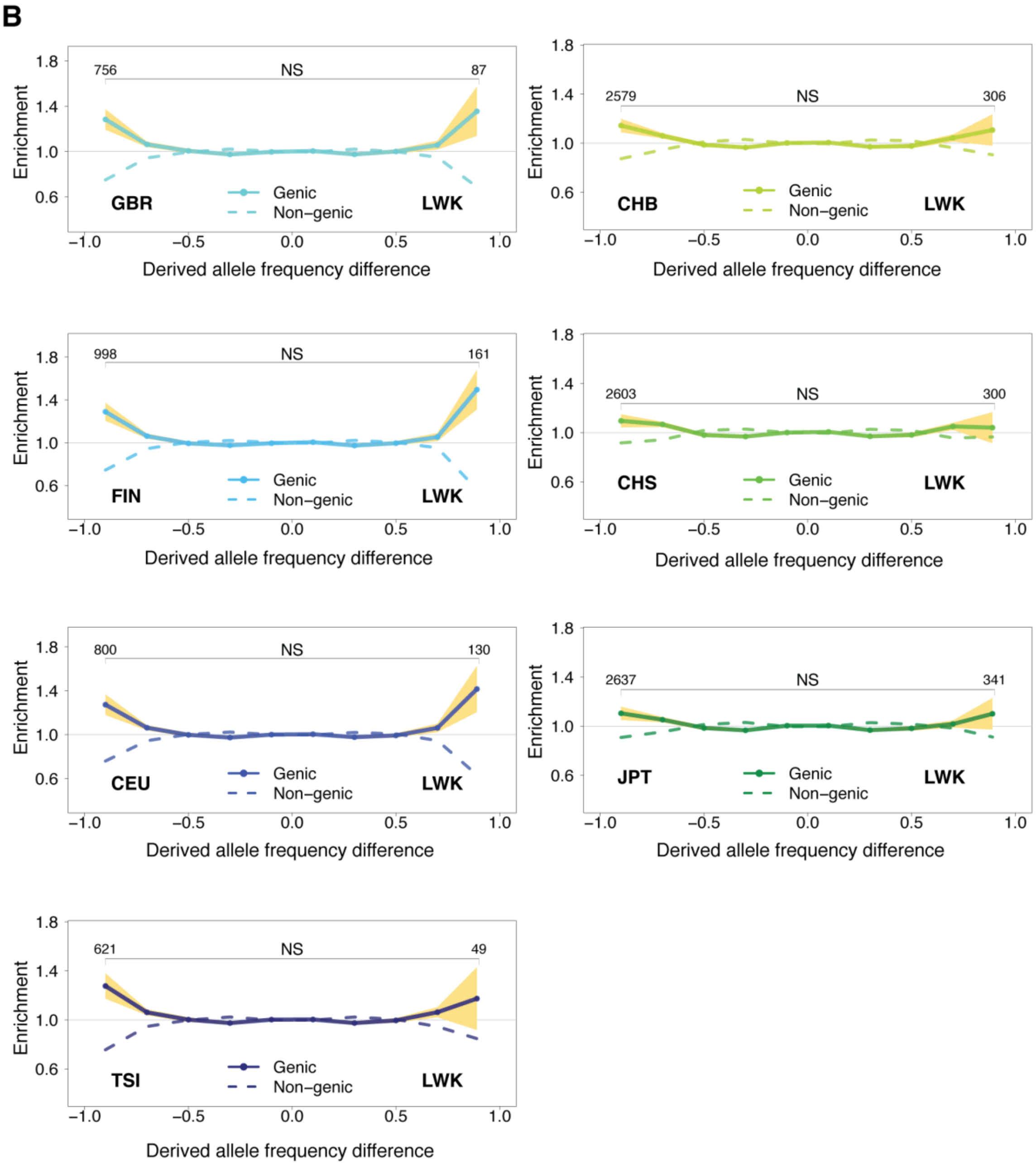

Supplementary Figure 3. Analyses of present-day population differentiation using weighted block jackknife. (**A**, **B**) Enrichment of strongly differentiated genic alleles for all present-day population comparisons using all European and East Asian populations versus (**A**) YRI or (**B**) LWK. The 95% confidence interval is shown in yellow based on a weighted block jackknife with 200kb genomic blocks. The level of significance of the bias in genic enrichment when comparing the two tails is shown on top (* < 0.05, ** < 0.01, and *** < 0.001, and NS for non-significant). The number of genic SNPs in each tail is also shown.

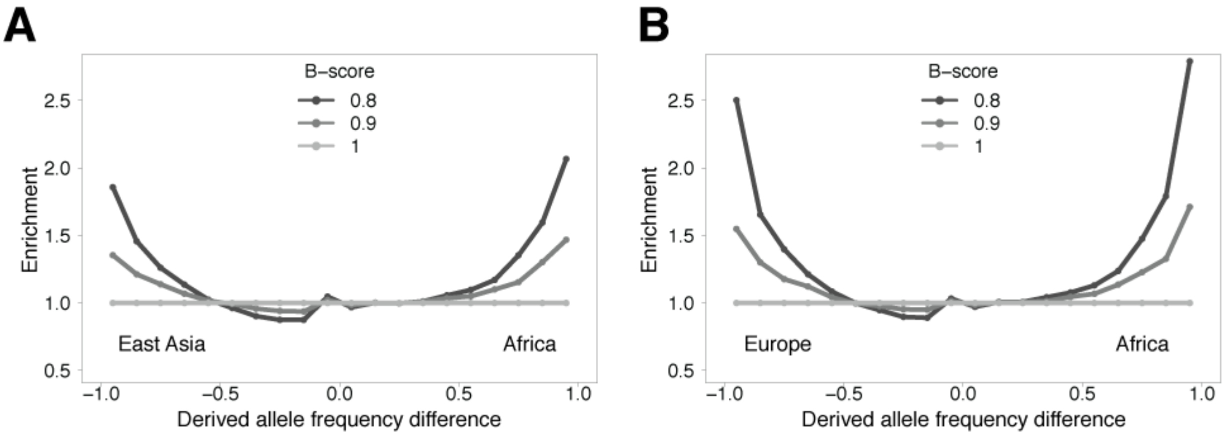

Supplementary Figure 4. Expectations under background selection alone. Expectation of genic enrichment in the absence of adaptive forces but with growing strengths of background selection (measured by lower B scores), based on coalescent simulations in the absence of positive selection for (**A**) East Asian and (**B**) European demography. See Methods about the use of 0.8 and 0.9 as B scores.

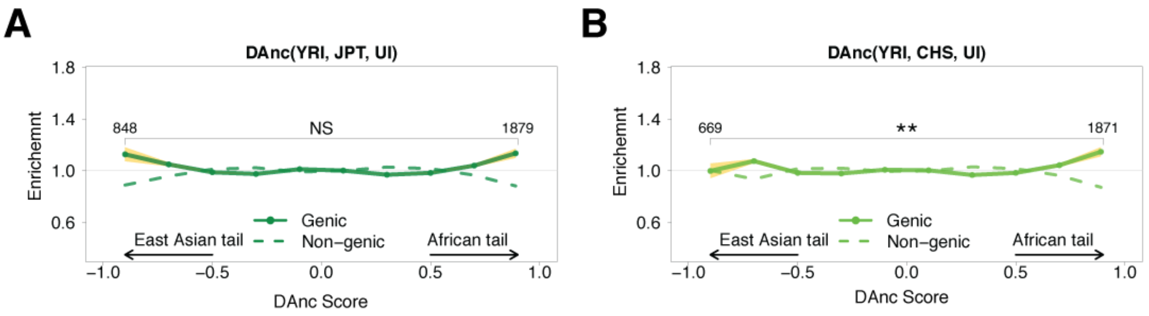

Supplementary Figure 5. Genic enrichment in other East Asian populations. Results of the enrichment analysis for the DAnc (YRI, P_2, Ust'-Ishim) analysis with (**A**) JPT or (**B**) CHS as P_2. The bootstrap 95% confidence interval is shown in yellow, and the level of significance of the bias in genic enrichment when comparing the two tails is shown on top (* < 0.05, ** < 0.01, and *** < 0.001, and NS for non-significant). The number of genic alleles in the tails is also shown.

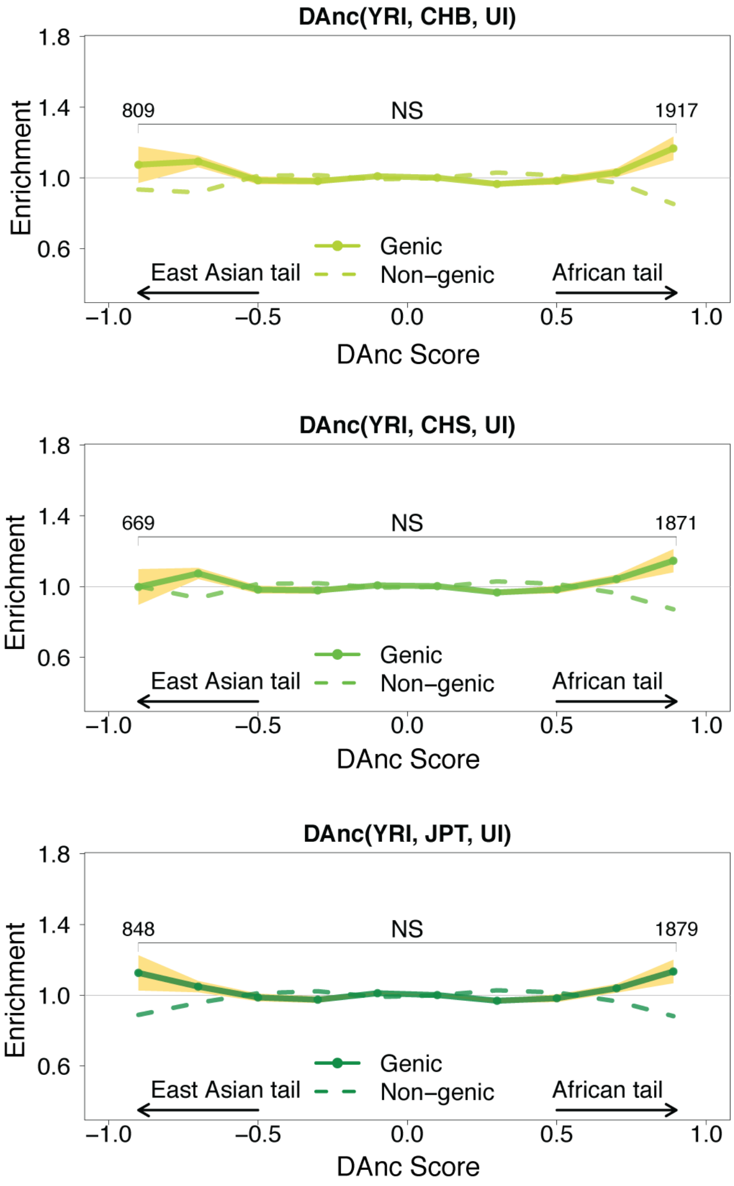

Supplementary Figure 6. Genic enrichment in East Asian populations using weighted block jackknife. Results of the enrichment analysis for the DAnc (YRI, P_2, Ust'-Ishim) analysis with CHB, CHS or JPT as P_2. The 95% confidence interval is shown in yellow based on a weighted block jackknife with 200kb genomic blocks. The level of significance of the bias in genic enrichment when comparing the two tails is shown on top (* < 0.05, ** < 0.01, and *** < 0.001, and NS for non-significant). The number of genic alleles in the tails is also shown.

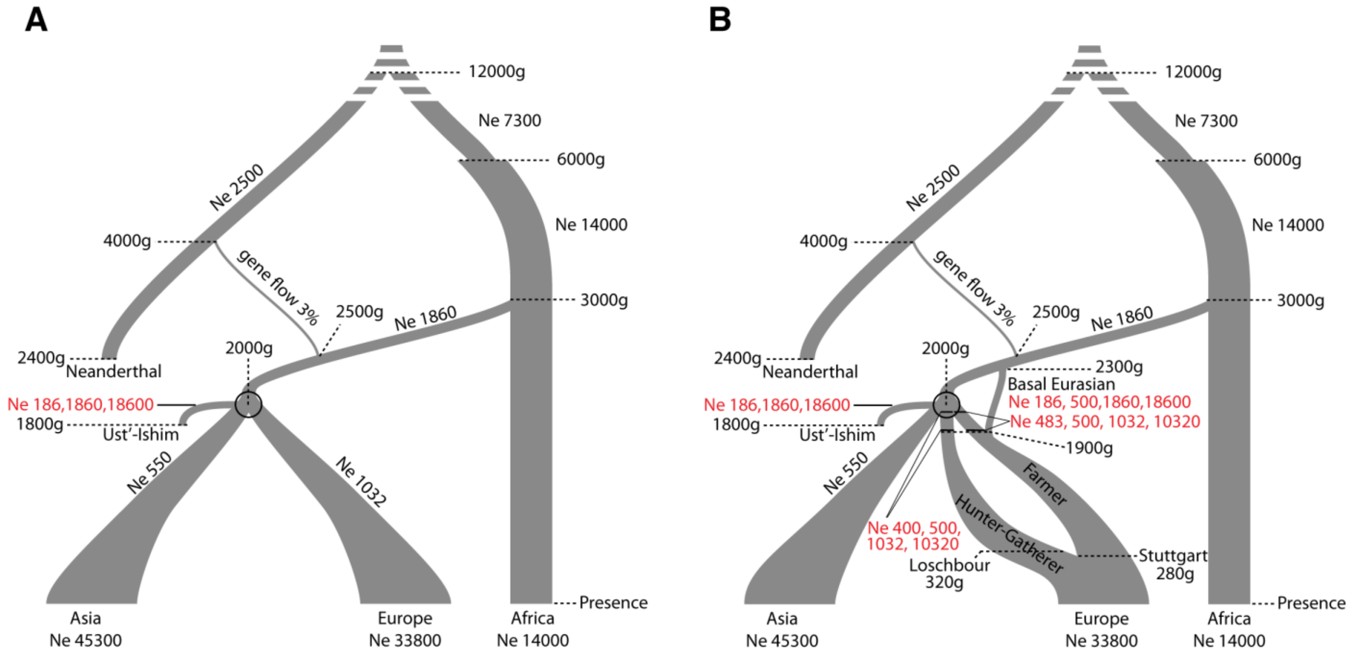

Supplementary Figure 7. Demographic parameters used for the simulations. The demographic parameters used for the simulations of present-day human populations are based on Gravel et al.[2]; parameters that we varied in different sets of simulations are marked in red (see Methods). (**A**) The demographic model used for simulations that includes the Ust'-Ishim population and sampling of the Ust'-Ishim genome. The Ust'-Ishim population is introduced in the Gravel[2] model following the model proposed by Fu et al.[3]. (**B**) The demographic model used for simulations that include the Ust'-Ishim and ancient European populations, and the sampling of Ust'-Ishim, a farmer (Stuttgart) and a hunter-gatherer (Loschbour). The ancient European populations were introduced in the Gravel[4] model following the model proposed by Lazaridis et al.[5]. Farmer and Basal Eurasian populations merge with proportions 56% / 44% [5]. Farmer and Hunter-Gatherer populations merge with proportion 50% / 50%. These proportions likely vary across European populations, but the DAnc European pattern observed is consistent across European populations (GBR, FIN, TSI, CEU). *g* stands for generations (25yrs).

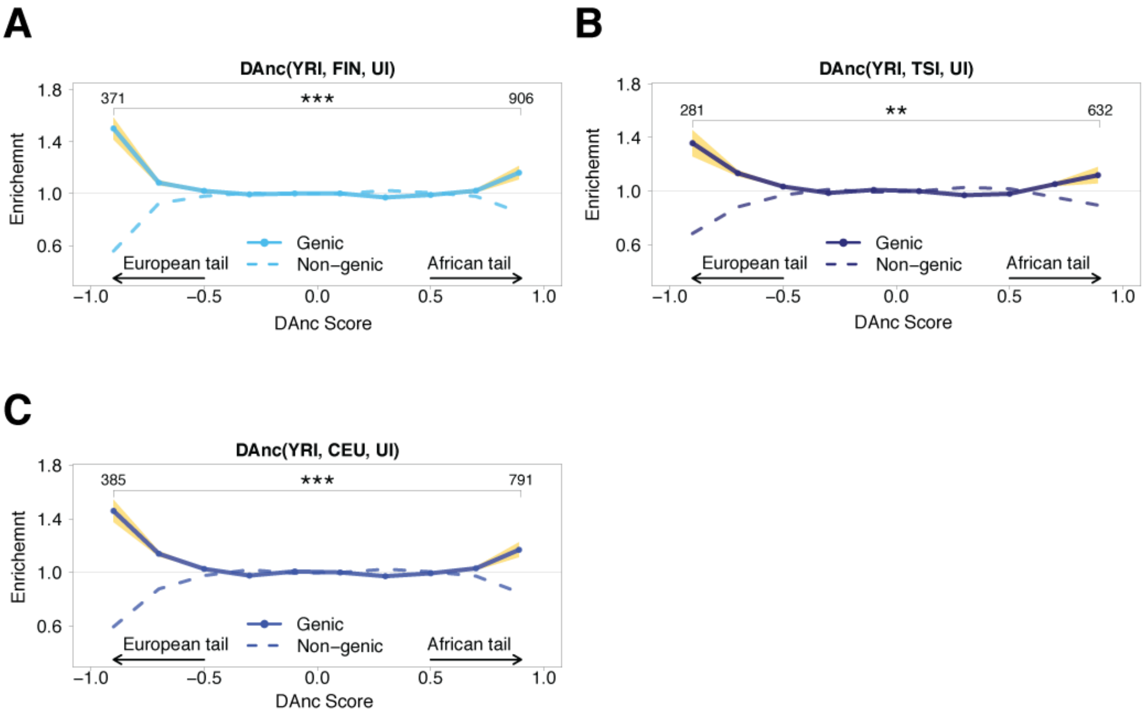

Supplementary Figure 8. Genic enrichment in other European populations. Results of the enrichment analysis for the DAnc (YRI, P_2, Ust'-Ishim) analysis with (**A**) FIN, (**B**) TSI, or (**C**) CEU as P_2**.** The bootstrap 95% confidence interval is shown in yellow, and the level of significance of the bias in genic enrichment when comparing the two tails is shown on top (* < 0.05, ** < 0.01, and *** < 0.001, and NS for non-significant). The number of genic alleles in the tails is also shown.

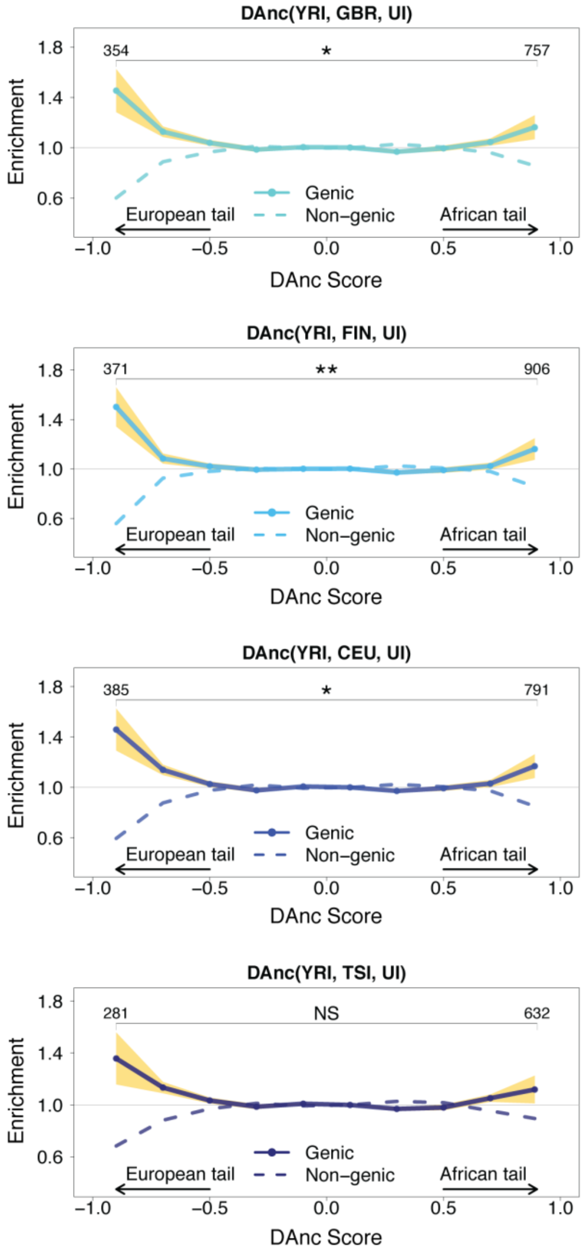

Supplementary Figure 9. Genic enrichment in European populations using weighted block jackknife. Results of the enrichment analysis for the DAnc (YRI, P_2, Ust'-Ishim) analysis with GBR, FIN, CEU or TSI as P_2. The 95% confidence interval is shown in yellow based on a weighted block jackknife with 200kb genomic blocks. The level of significance of the bias in genic enrichment when comparing the two tails is shown on top (* < 0.05, ** < 0.01, and *** < 0.001, and NS for non-significant). The number of genic alleles in the tails is also shown. The enrichment in genic alleles in significantly stronger in the European tail for all populations except TSI, which has been inferred to have low levels of hunter-gatherer ancestry[6,7]. Our results show that this ancestral component is relevant to this analysis (discussed in detail in the *Highly differentiated alleles in ancient Europeans* section of the main text).

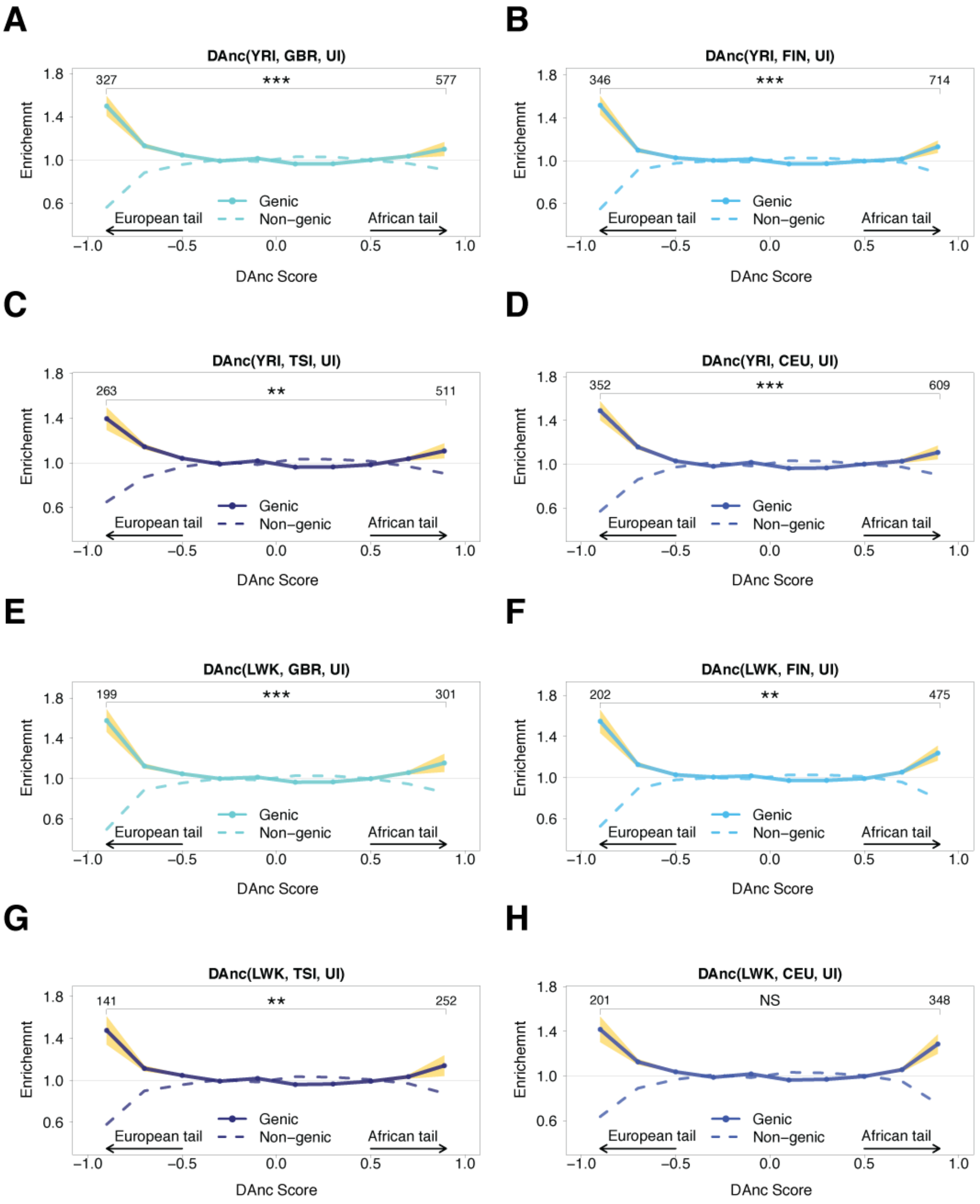

Supplementary Figure 10. Genic enrichment in European populations versus YRI or LWK using only sites with higher derived allele frequency in the European population. Results of the enrichment analysis for DAnc (P_1, P_2, Ust'-Ishim) analysis with (**A-D**) YRI and (**E-H**) LWK as P_1, and (**A,E**) GBR, (**B,F**), FIN, (**C,G**) TSI, or (**D,H**) CEU as P_2. The bootstrap 95% confidence interval is shown in yellow, and the level of significance of the bias in genic enrichment when comparing the two tails is shown on top (* < 0.05, ** < 0.01, and *** < 0.001, and NS for non-significant). The number of genic alleles in the tails is also shown.

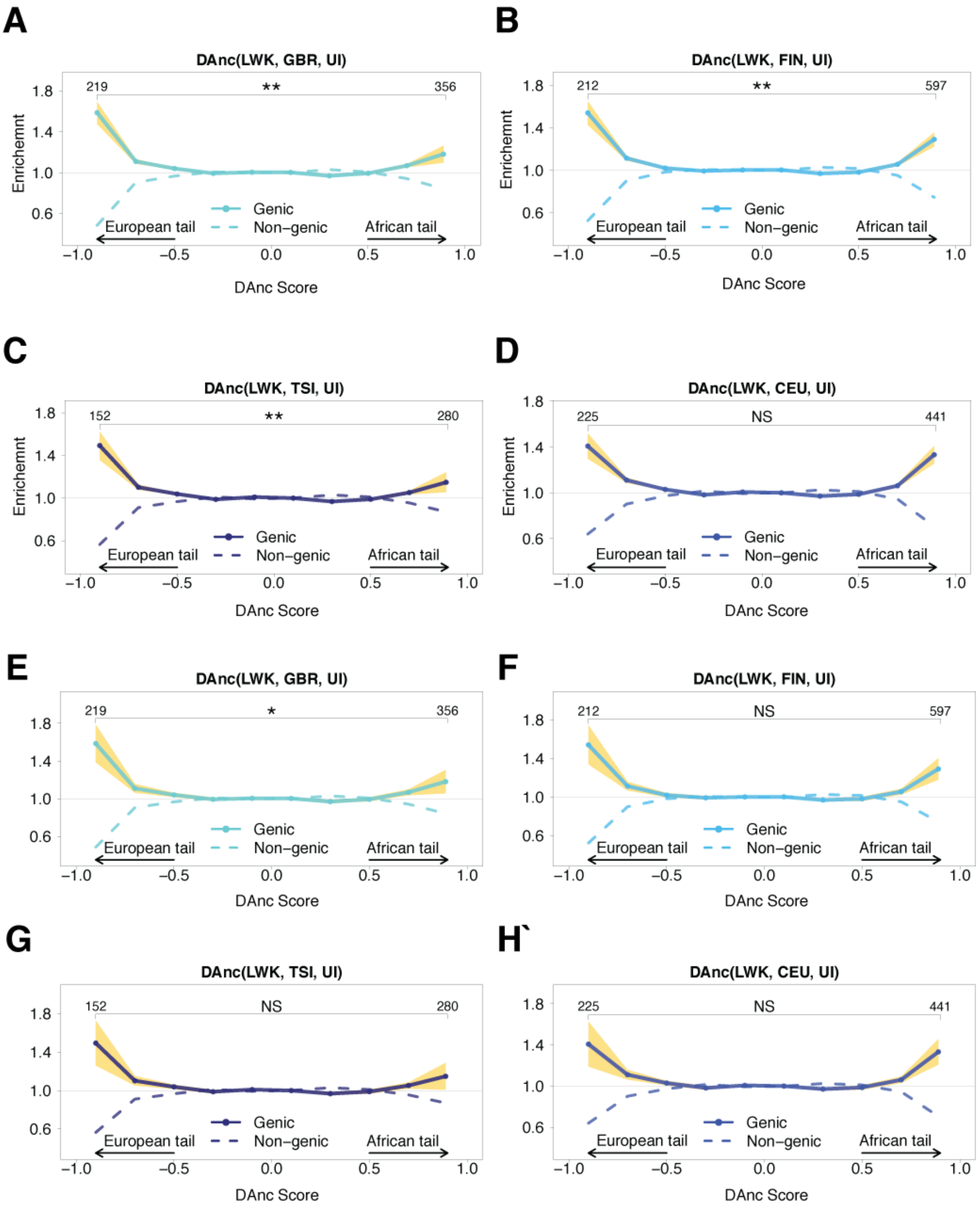

Supplementary Figure 11. Genic enrichment in European populations versus LWK (Luhya, East Africa)**.** Results of the enrichment analysis for DAnc (LWK, P_2, Ust'-Ishim) analysis with (**A, E**) GBR, (**B, F**), FIN, (**C, G**) TSI, or (**D, H**) CEU as P_2. In yellow (**A-D**) shows the bootstrap 95% confidence interval and (**E-H**) the 95% confidence interval based on a weighted block jackknife with 200kb genomic blocks. The level of significance of the bias in genic enrichment when comparing the two tails is shown on top (* < 0.05, ** < 0.01, and *** < 0.001, and NS for non-significant). The number of genic alleles in the tails is also shown.

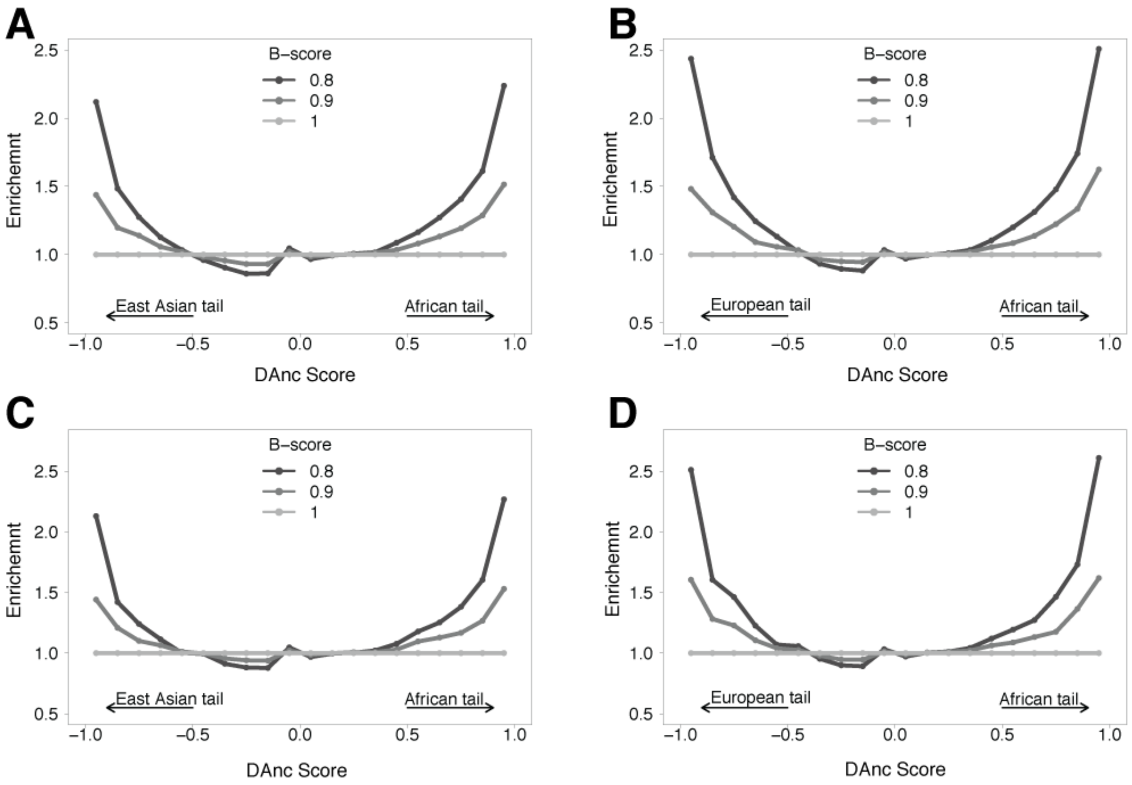

Supplementary Figure 12. Effects of variation in Ust'-Ishim Ne for non-adaptive simulations. Enrichment analyses with background selection (measured by B scores) and without background selection (B = 1) under our (**A, C**) East Asian or (**B, D**) European demographic model with (**A, C**) Ust'-Ishim Ne=186, or (**B, D**) Ust'-Ishim Ne=18600.

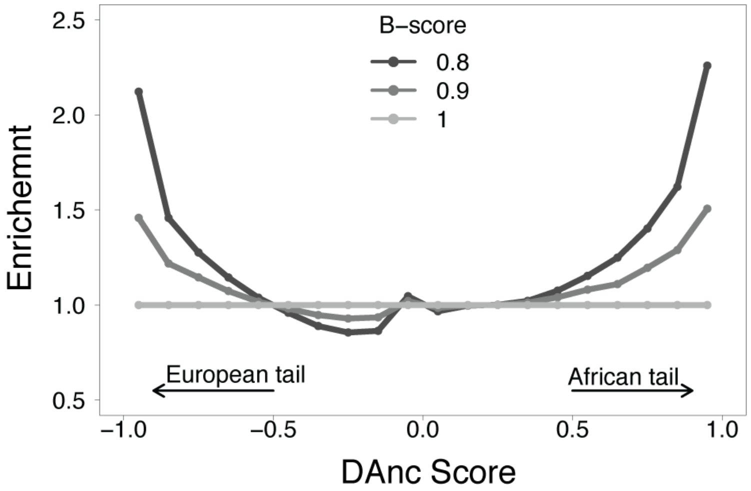

Supplementary Figure 13. Expectations under background selection alone for more complex European demography. Expectation of genic enrichment using DAnc in the absence of adaptive forces but with growing strengths of background selection (measured by lower B scores), based on coalescent simulations without positive selection for European demography (including basal Eurasian, hunter-gatherer and farmer populations, see Figure 2B, Supplementary Fig. 7 and Methods). See Methods about the use of 0.8 and 0.9 as B scores.

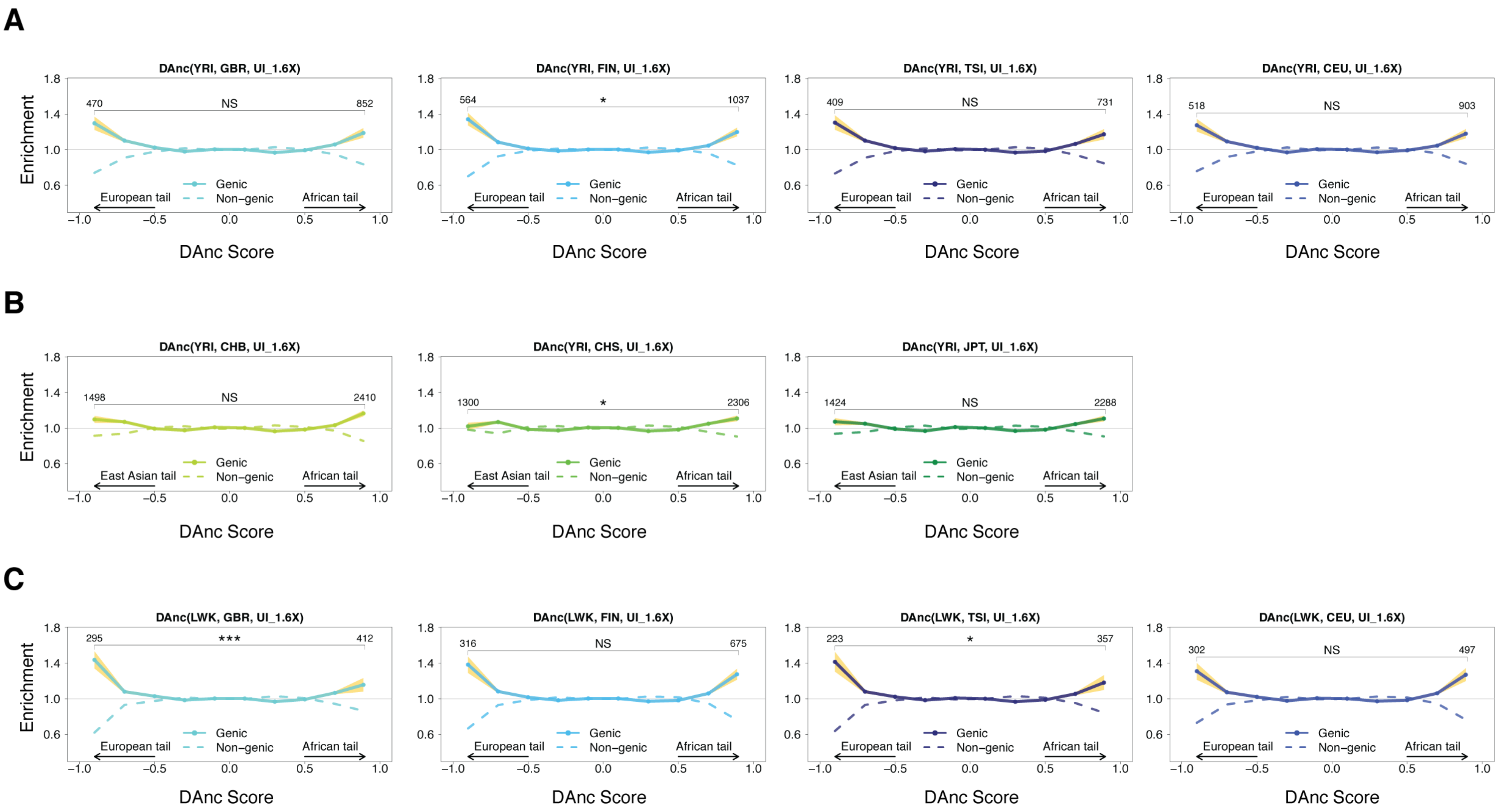
A
DAnc(YRI, GBR, UI_1.6X)
470
NS
852
DAnc(YRI, FIN, UI_1.6X)
564
*
1037
DAnc(YRI, TSI, UI_1.6X)
409
NS
731
DAnc(YRI, CEU, UI_1.6X)
518
NS
903
B
DAnc(YRI, CHB, UI_1.6X)
1498
NS
2410
DAnc(YRI, CHS, UI_1.6X)
1300
*
2306
DAnc(YRI, JPT, UI_1.6X)
1424
NS
2288
C
DAnc(LWK, GBR, UI_1.6X)
295
***
412
DAnc(LWK, FIN, UI_1.6X)
316
NS
675
DAnc(LWK, TSI, UI_1.6X)
223
*
357
DAnc(LWK, CEU, UI_1.6X)
302
NS
497
Enrichment
DAnc Score
Genic
Non−genic
European tail
East Asian tail
African tail

D

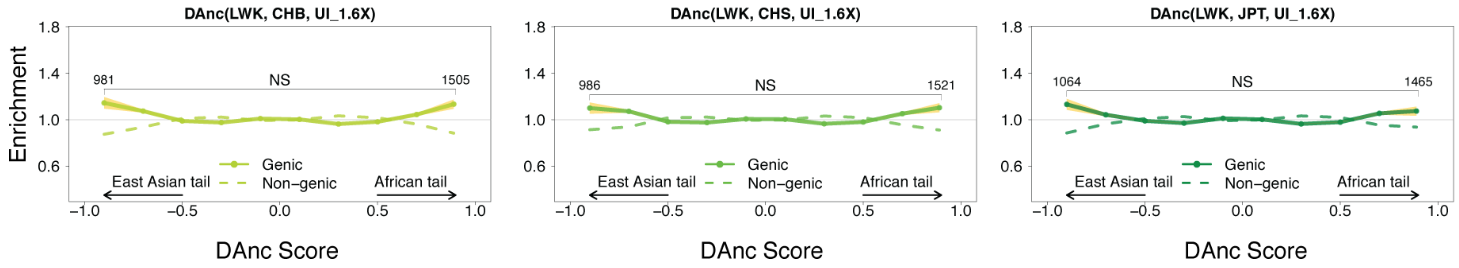

Supplementary Figure 14. DAnc analysis using low coverage Ust'-Ishim (1.6X). Genic enrichment in alleles in different bins of the DAnc statistic (P_1, P_2, Ust'-Ishim). (**A**) P_1 is YRI and P_2 all European populations, (**B**) P_1 is YRI and P_2 all East Asian populations, (**C**) P_1 is LWK and P_2 all European populations, and (**D**) P_1 is LWK and P_2 all East Asian populations. The bootstrap 95% confidence interval is shown in yellow, and the level of significance of the bias in genic enrichment when comparing the two tails is shown on top (* < 0.05, ** < 0.01, and *** < 0.001, and NS for non-significant). The number of genic sites in each tail is on top of the tails. See methods for details on the low-coverage Ust'-Ishim genotypes.

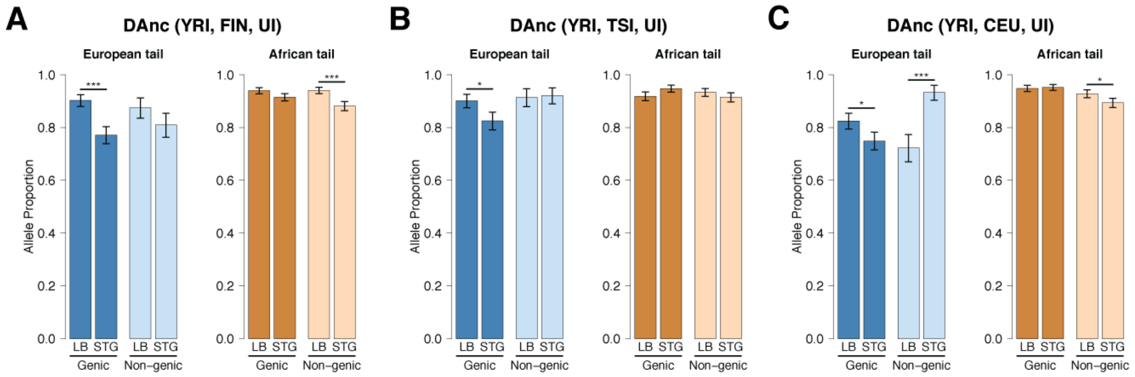

Supplementary Figure 15. Presence in ancient European genomes of the high-frequency European allele in alleles in the DAnc European and African tail. Proportion of European alleles present in Loschbour (LB) or Stuttgart (STG) among those in the tails of the DAnc (YRI, P_2, Ust'-Ishim) distribution using (**A**) FIN, (**B**) TSI, and (**C**) CEU as P_2. The bootstrap 95% confidence interval is shown and asterisks indicate significant differences between LB and STG (* < 0.05, ** < 0.01, and *** < 0.001). GBR is presented in Figure 4A.

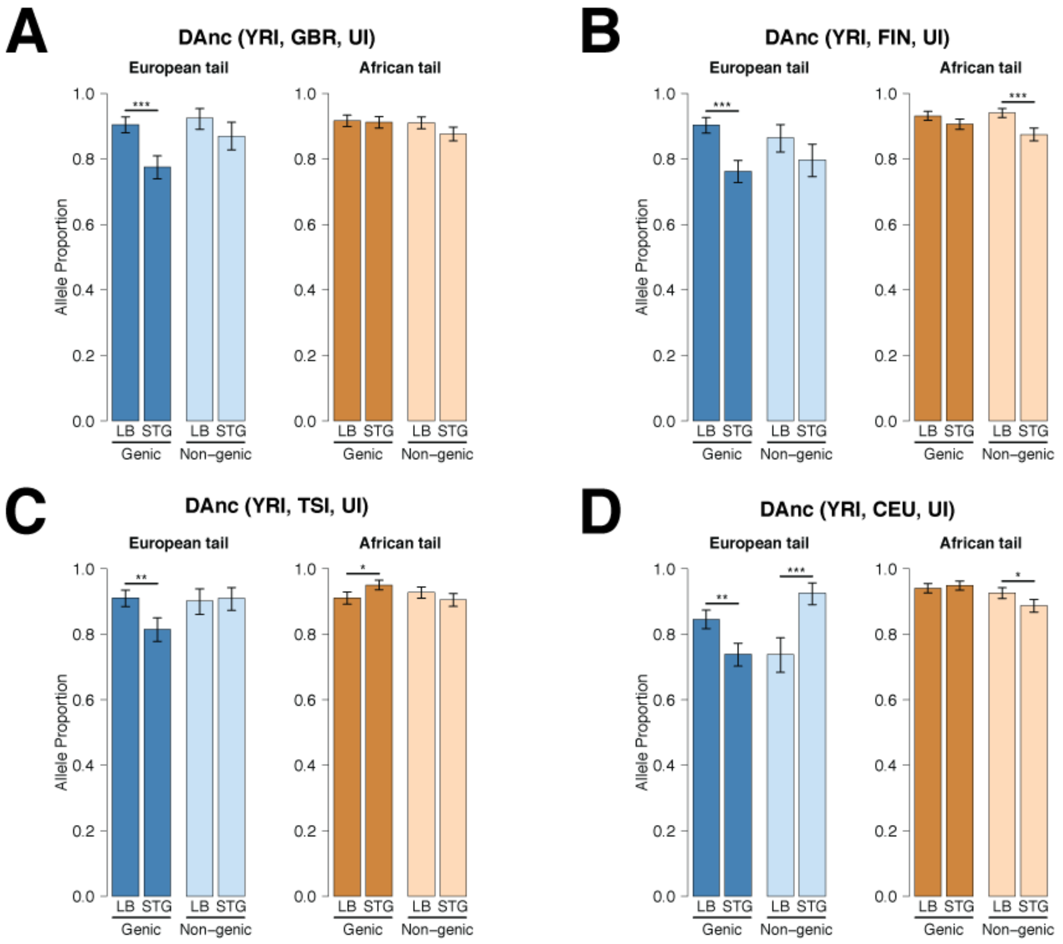

Supplementary Figure 16. Presence in ancient European genomes of the high-frequency European allele in alleles in the DAnc European and African tail (using only sites where the derived allele reached high frequency in Europe). Proportion of European alleles present in Loschbour (LB) or Stuttgart (STG) among those in the tails of the DAnc (YRI, P_2, Ust'-Ishim) distribution using (**A**) GBR, (**B**) FIN, (**C**) TSI, and (**D**) CEU as P_2. The bootstrap 95% confidence interval is shown and asterisks indicate significant differences between LB and STG (* < 0.05, ** < 0.01, and *** < 0.001).

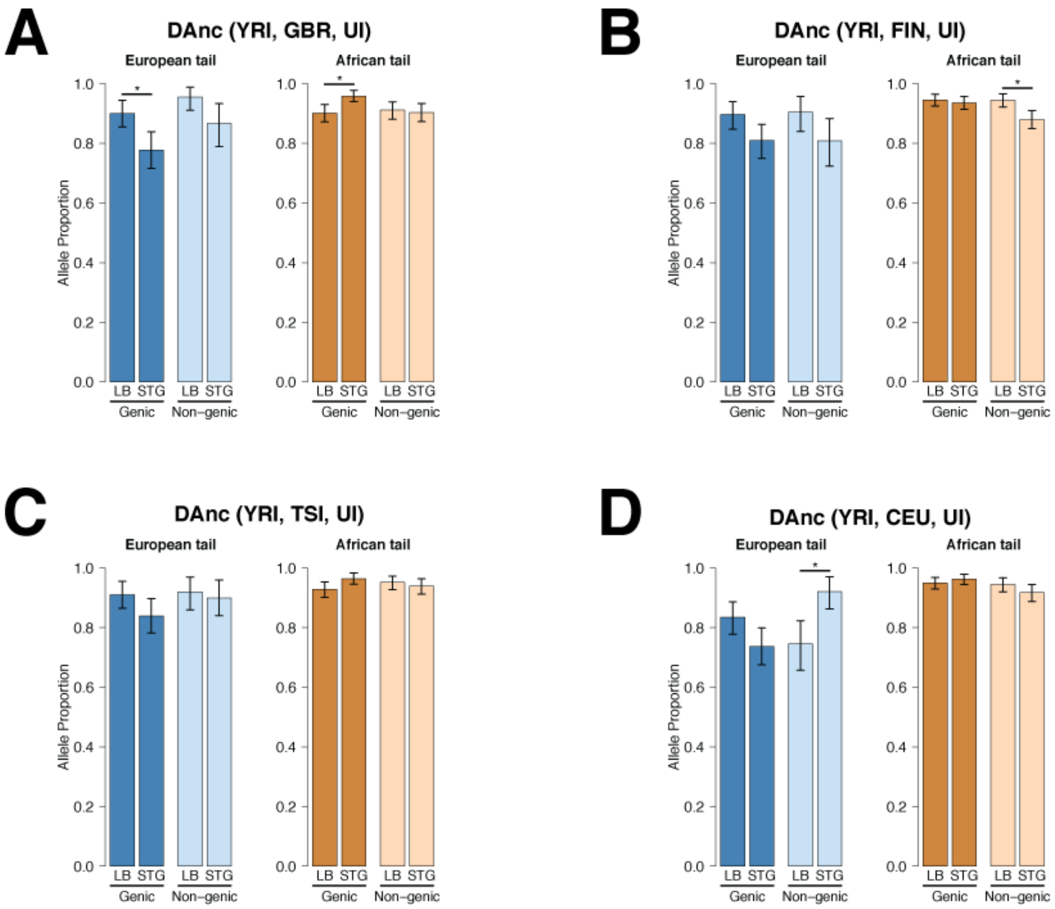

Supplementary Figure 17. Presence in ancient European genomes of the high-frequency European allele in alleles in the DAnc European and African tail (using only transversions). Proportion of European alleles present in Loschbour (LB) or Stuttgart (STG) among those in the tails of the DAnc (YRI, P_2, Ust'-Ishim) distribution using (**A**) GBR, (**B**) FIN, (**C**) TSI, and (**D**) CEU as P_2. The bootstrap 95% confidence interval is shown and asterisks indicate significant differences between LB and STG (* < 0.05, ** < 0.01, and *** < 0.001).

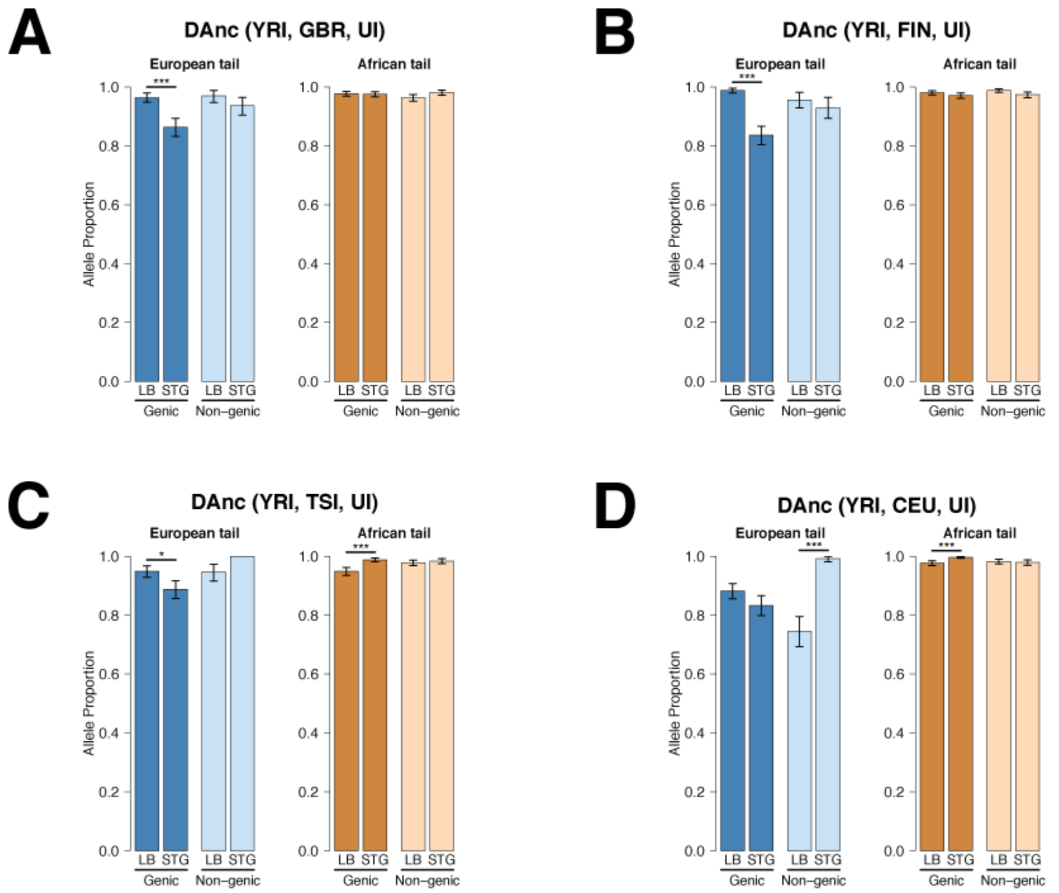

Supplementary Figure 18. Presence in ancient European genomes of the high-frequency European allele in alleles in the DAnc European and African tail (using only sites that are homozygous in the ancient European genomes). Proportion of European alleles present in Loschbour (LB) or Stuttgart (STG) among those in the tails of the DAnc (YRI, P_2, Ust'-Ishim) distribution using (**A**) GBR, (**B**) FIN, (**C**) TSI, and (**D**) CEU as P_2. The bootstrap 95% confidence interval is shown and asterisks indicate significant differences between LB and STG (* < 0.05, ** < 0.01, and *** < 0.001).

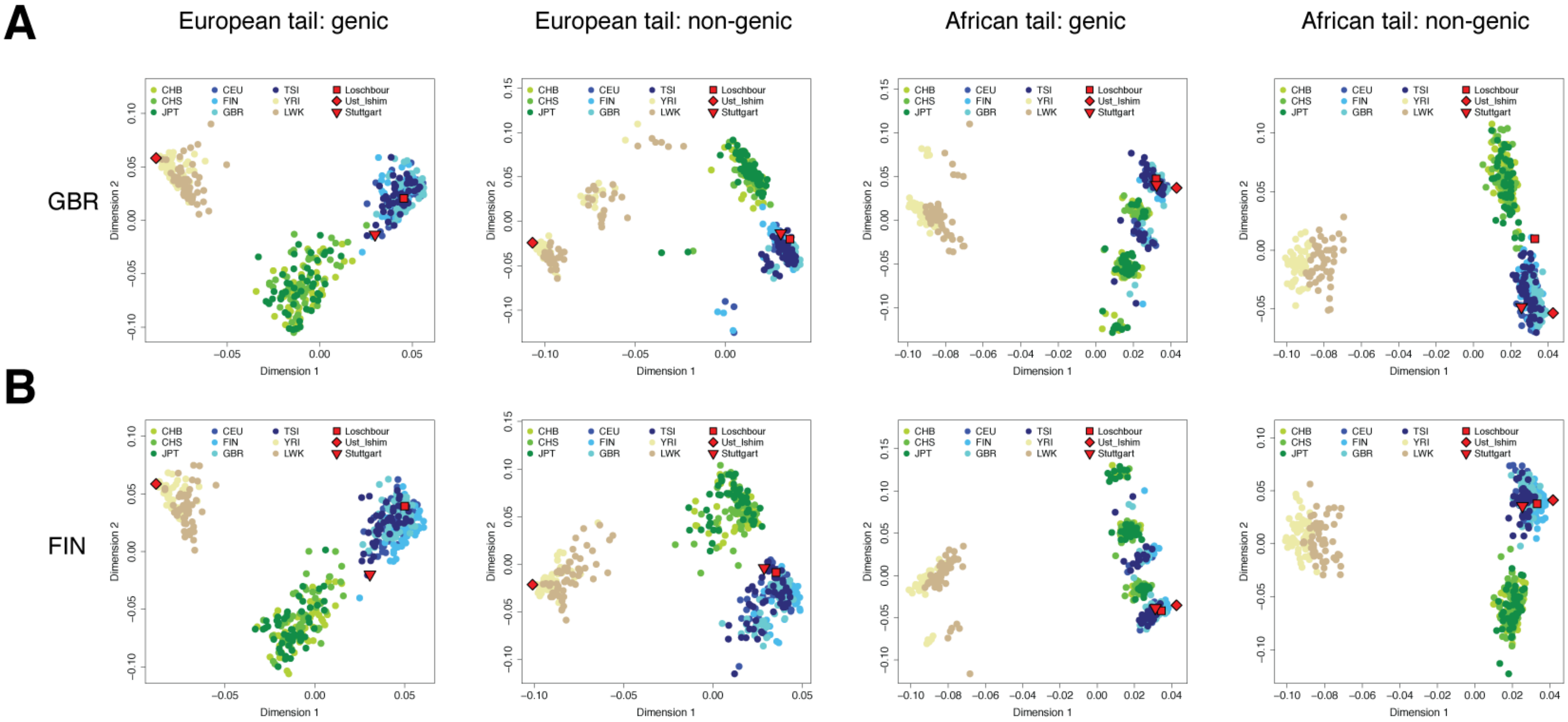
A
B
European tail: genic
European tail: non-genic
African tail: genic
African tail: non-genic
GBR
FIN
CHB
CHS
JPT
CEU
FIN
GBR
TSI
YRI
LWK
Loschbour
Ust_Ishim
Stuttgart
Dimension 1
Dimension 2

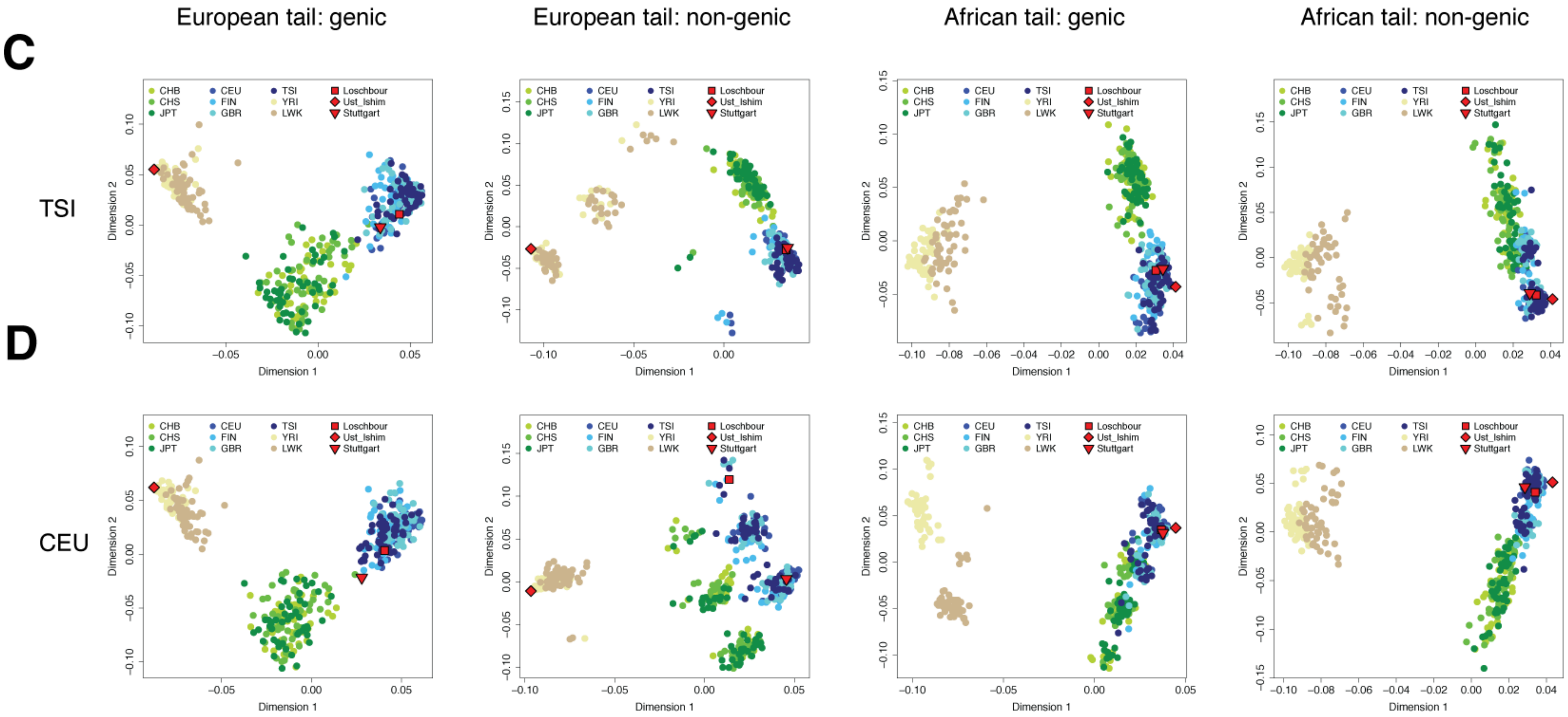

Supplementary Figure 19. Principal component analysis of present-day human populations and ancient genomes (Ust'-Ishim, Loschbour, Stuttgart). PCA of all alleles in the African and European tail of the DAnc (YRI, P_2, Ust'-Ishim) distribution for (**A**) GBR, (**B**) FIN, (**C**) TSI, and (**D**) CEU as P_2. The present-day populations are color-coded and the ancient Ust'-Ishim, Loschbour, and Stuttgart samples are projected into the present-day human dataset. The PCA is based on the pairwise allele sharing distances[8] among the present-day humans and a principal components analysis (PCA) on the resulting pairwise distance matrix, with projected genotyping calls from the ancient samples onto the PCA using SMARTPCA[9].

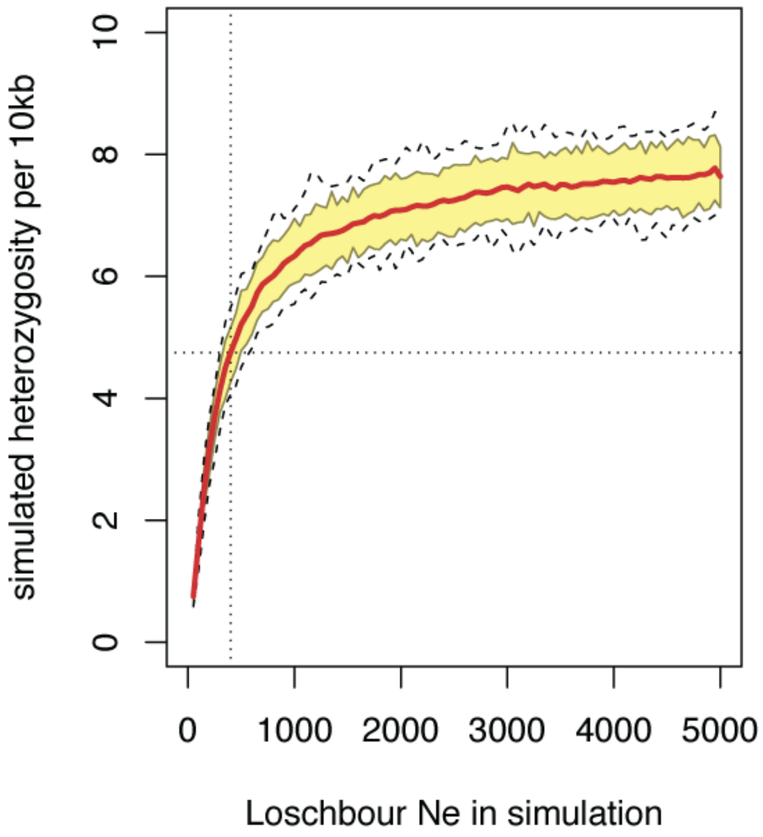

Supplementary Figure 20. Inference of Ne in Loschbour. For each putative Ne of Loschbour (steps of 50) we ran 100,000 simulations under our (otherwise) demographic model. The red curve shows the mean heterozygosity (*H*), the yellow area the 95% confidence interval of *H*, and the dashed lines the minimum and maximum *H* values from these simulations for Loschbour. The dotted horizontal line indicates the observed *H* (mlrho) in the real data[5] and the vertical line the intercept with the simulated *H*.

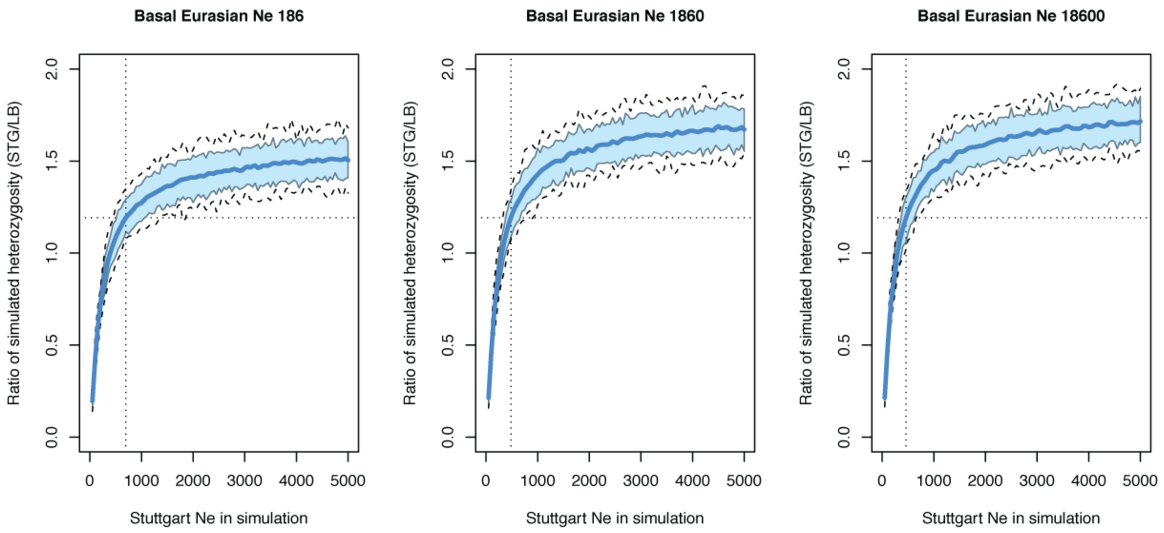

Supplementary Figure 21. Inference of Ne in Stuttgart using different Ne in the basal Eurasian population (Ne equals 186, 1860, or 18600).
For each putative Ne of Stuttgart (steps of 50) we ran 100,000 simulations under our (otherwise) demographic model. The blue curve shows the mean heterozygosity (*H*), the light blue area the 95% confidence interval of *H*, and the dashed lines the minimum and maximum *H* values from these simulations for Stuttgart. The dotted horizontal line indicates the observed ratio of transversions (H_{STG} / H_{LB} = 1.19) in the real data and the vertical line its intercept with the simulated H_{STG} / H_{LB} ratio.

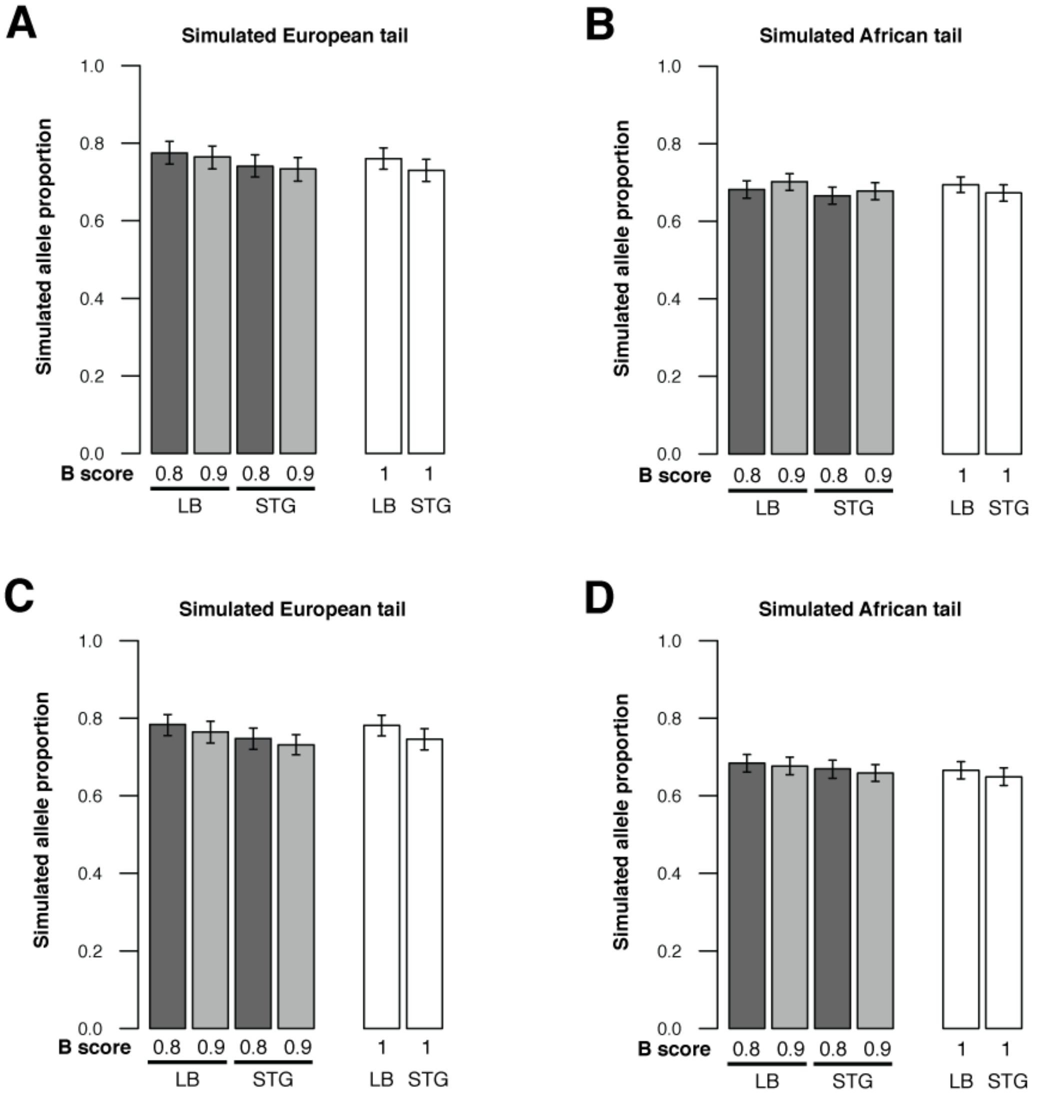

Supplementary Figure 22. **Expectation of the proportion of the high-frequency European alleles** in Loschbour (LB) and Stuttgart (STG) for alleles in the (**A, C**) European and the (**B, D**) African tail of the DAnc analysis, when the Ne of the basal Eurasian population is (**A, B**) 186 or (**C, D**) 18,600. The bootstrap 95% confidence interval shown is obtained.

A

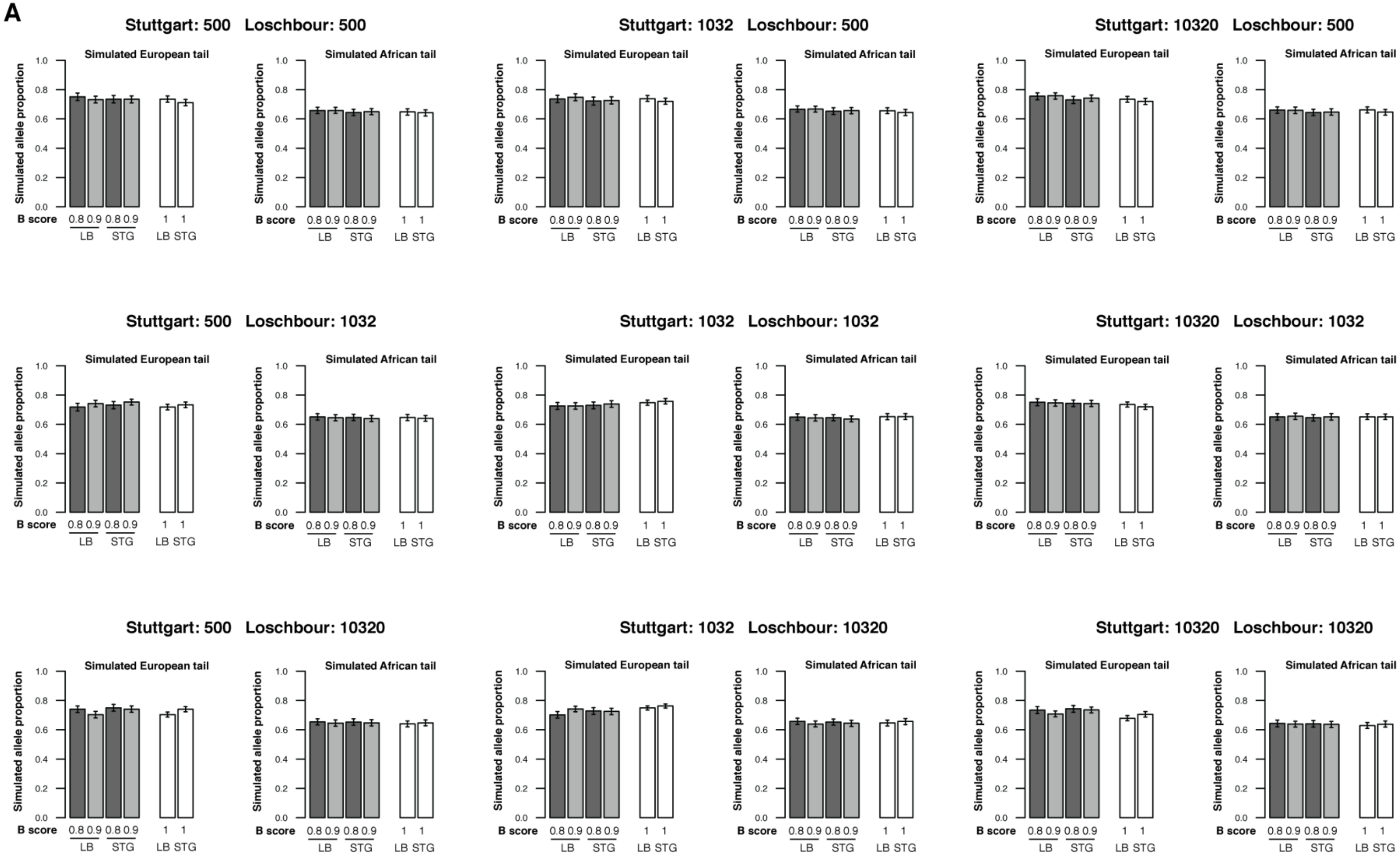
Stuttgart: 500 Loschbour: 500
Stuttgart: 1032 Loschbour: 500
Stuttgart: 10320 Loschbour: 500
Stuttgart: 500 Loschbour: 1032
Stuttgart: 1032 Loschbour: 1032
Stuttgart: 10320 Loschbour: 1032
Stuttgart: 500 Loschbour: 10320
Stuttgart: 1032 Loschbour: 10320
Stuttgart: 10320 Loschbour: 10320
Simulated European tail
Simulated African tail
Simulated allele proportion
B score
0.8 0.9 0.8 0.9 1 1
LB STG LB STG

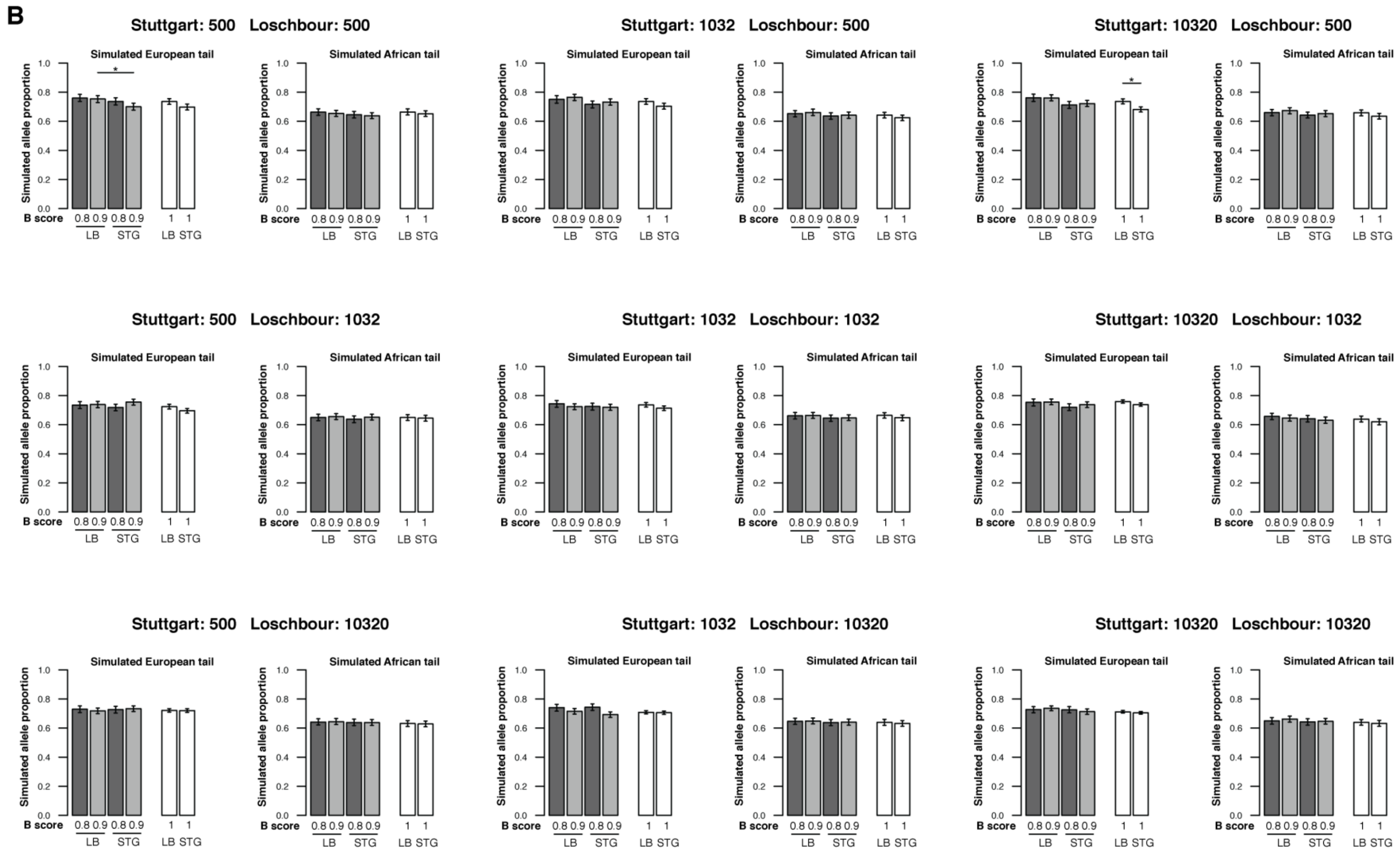

Supplementary Figure 23. Expectation of the proportion of the high-frequency European alleles under more extreme Ne values in ancient European populations. The Ne of the Basal Eurasians is set to (**A**) 500 and (**B**) 1860. For each, we ran simulations with a range of Ne for Loschbour and Stuttgart (500, 1032, 10320 respectively). The bootstrap 95% confidence interval is shown. One single simulation set is significant, but an enrichment of only 5.4% (much lower than the average 9.7% in real data).

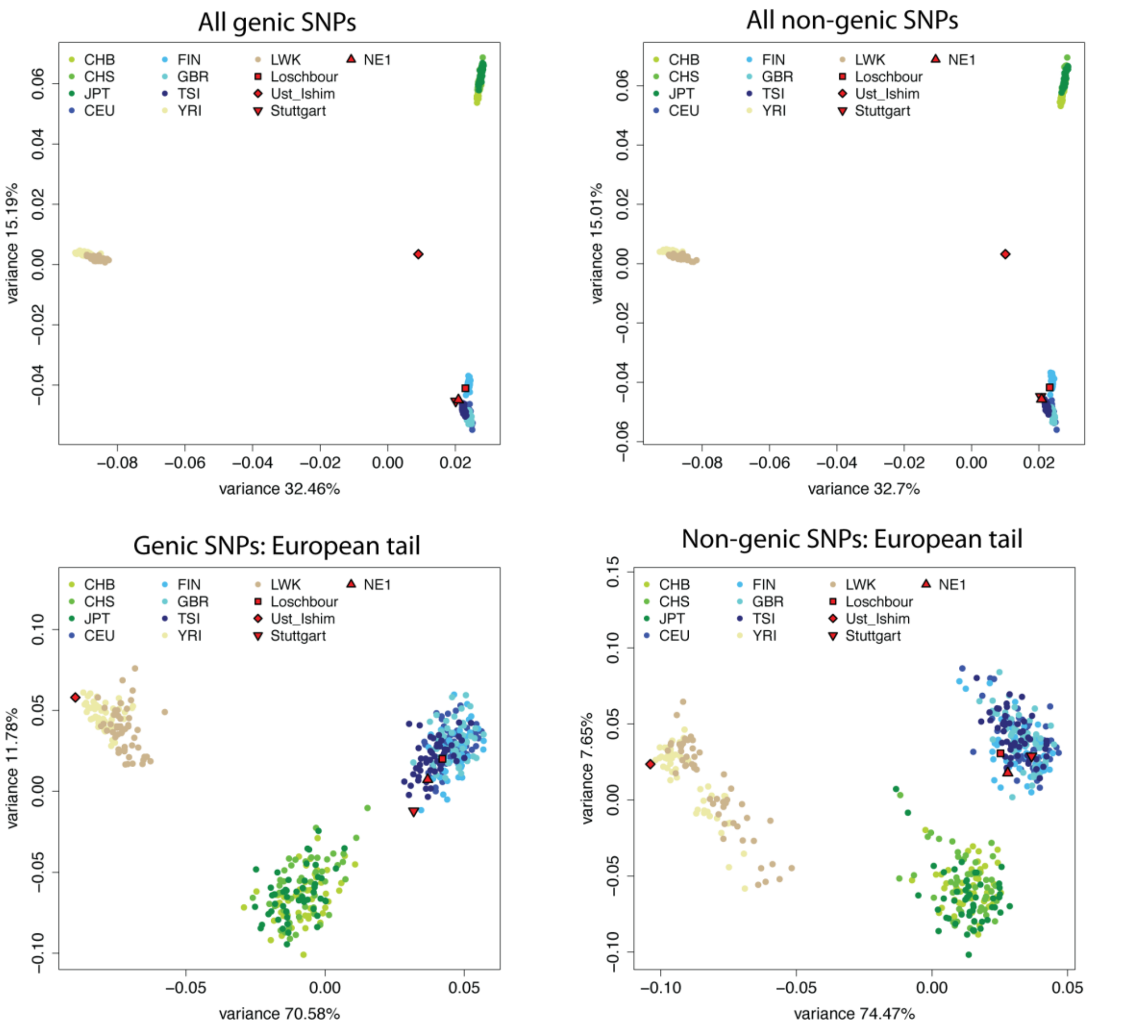

Supplementary Figure 24. Principal component analysis of present-day human populations and ancient genomes (Ust'-Ishim, Loschbour, Stuttgart, NE1). The present-day populations are color-coded and the ancient Ust'-Ishim, Loschbour, Stuttgart, and NE1 genomes are projected into the present-day human dataset. PCA's are shown for all genic and non-genic SNPs as well as the genic and non-genic DAnc European tails. The variance explained by PC1 and PC2 is indicated in the axis labels. The PCA is based on the pairwise allele sharing distances[8] among the present-day humans and a principal components analysis (PCA) on the resulting pairwise distance matrix, with projected genotyping calls from the ancient samples onto the PCA using SMARTPCA[9].

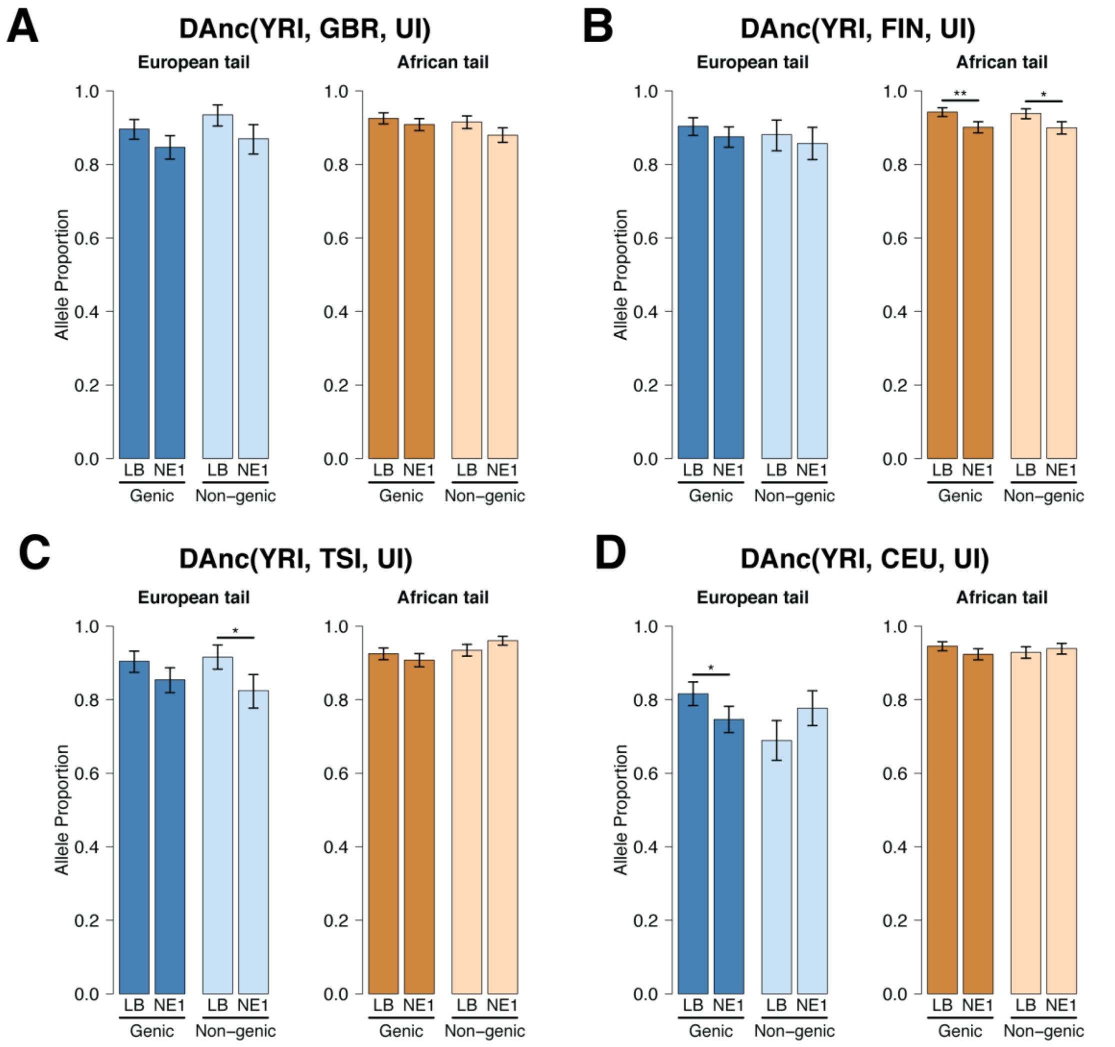

Supplementary Figure 25. Presence in ancient European genomes of the high-frequency European allele in alleles in the DAnc European and African tail. Proportion of European alleles present in Loschbour (LB) or NE1 among those in the tails of the DAnc (YRI, P_2, Ust'-Ishim) distribution using (**A**) GBR, (**B**) FIN, (**C**) TSI, and (**D**) CEU as P_2. The bootstrap 95% confidence interval is shown and asterisks indicate significant differences between LB and NE1 (* < 0.05, ** < 0.01, and *** < 0.001).

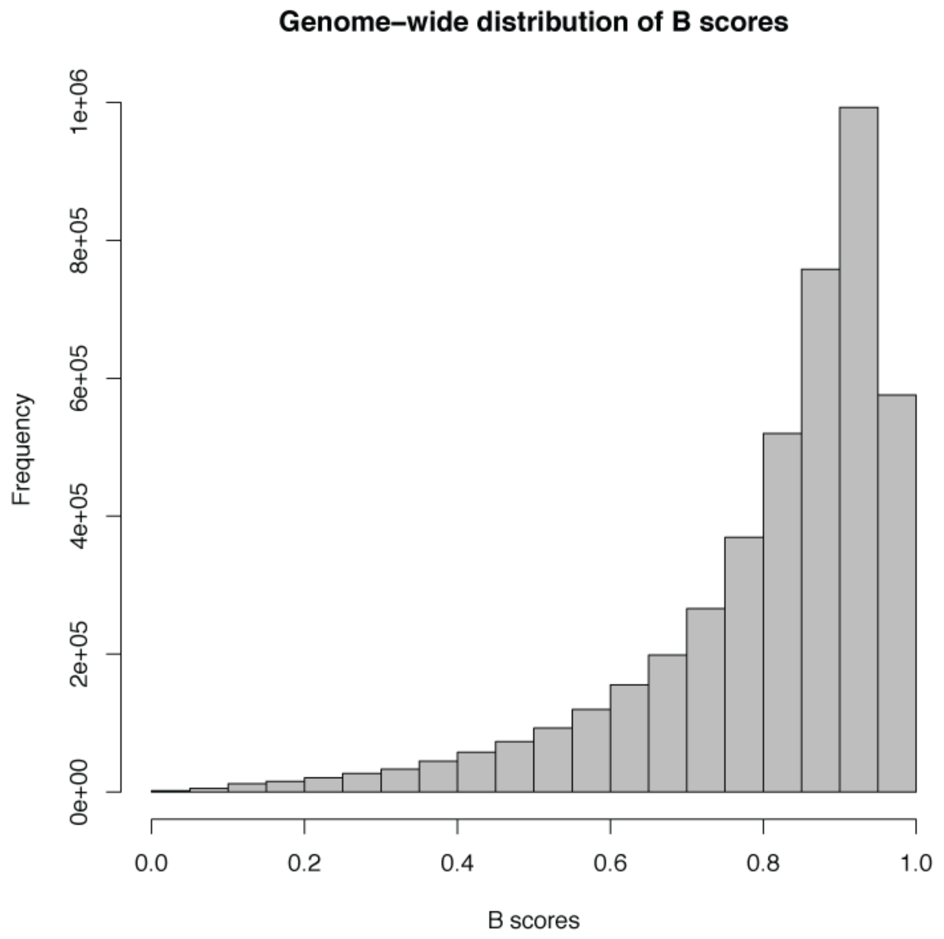

Supplementary Figure 26. Genome-wide distribution of B scores based on McVicker et al.

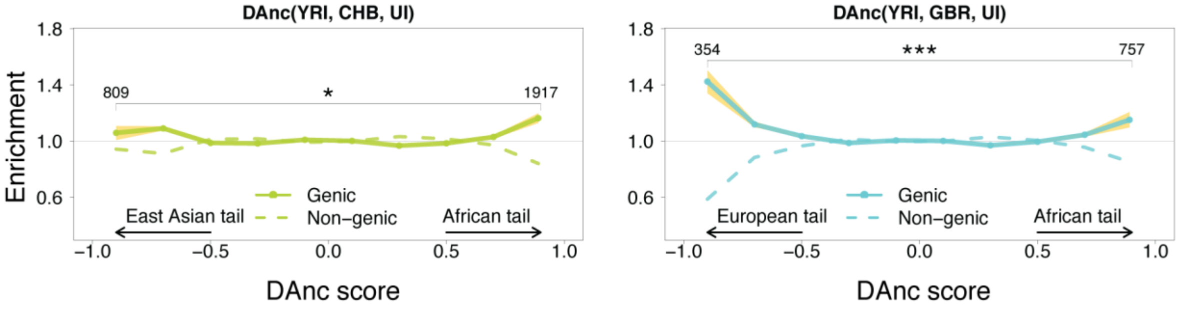

Supplementary Figure 27. Results of the enrichment analysis for DAnc(YRI, CHB, UI) and DAnc(YRI, GBR, UI) using the original genic and non-genic annotation. The non-genic category was downsampled to match the number of variants in the genic category (8,169,486 variants). The bootstrapped 95% confidence interval is shown in yellow, and the level of significance of the bias in genic enrichment when comparing the two tails is shown on top (* < 0.05, ** < 0.01, and *** < 0.001, and NS for non-significant). The number of genic sites in each tail is on top of the tails.

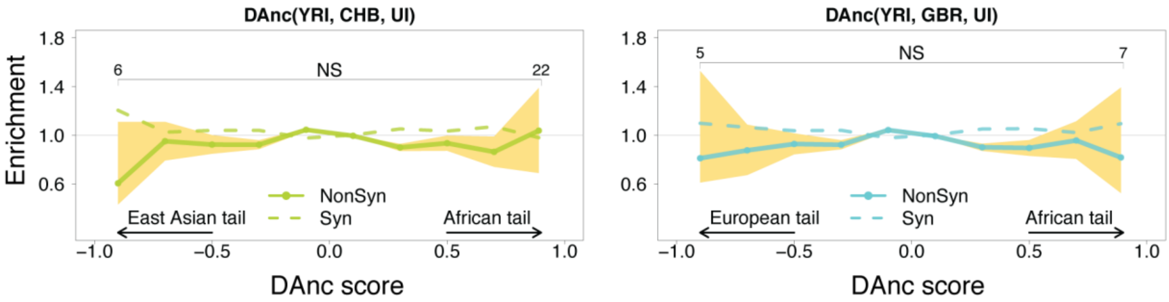

Supplementary Figure 28. Results of the enrichment analysis for DAnc(YRI, CHB, UI) and DAnc(YRI, GBR, UI) using as annotation synonymous and non-synonymous variants. The bootstrapped 95% confidence interval is shown in yellow, and the level of significance of the bias in genic enrichment when comparing the two tails is shown on top (* < 0.05, ** < 0.01, and *** < 0.001, and NS for non-significant). The number of genic sites in each tail is on top of the tails.

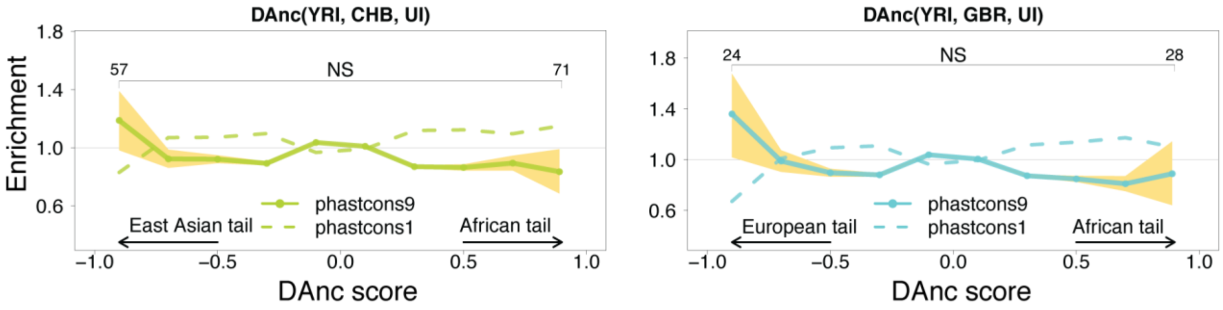

Supplementary Figure 29. Results of the enrichment analysis for DAnc(YRI, CHB, UI) and DAnc(YRI, GBR, UI) using as annotation conserved (phastCons > 0.9) and non-conserved (phastCons < 0.1) variants. The non-conserved category was downsampled to match the number of variants in the conserved category (516,448 variants). The bootstrapped 95% confidence interval is shown in yellow, and the level of significance of the bias in conserved enrichment when comparing the two tails is shown on top (* < 0.05, ** < 0.01, and *** < 0.001, and NS for non-significant). The number of conserved sites in each tail is on top of the tails.

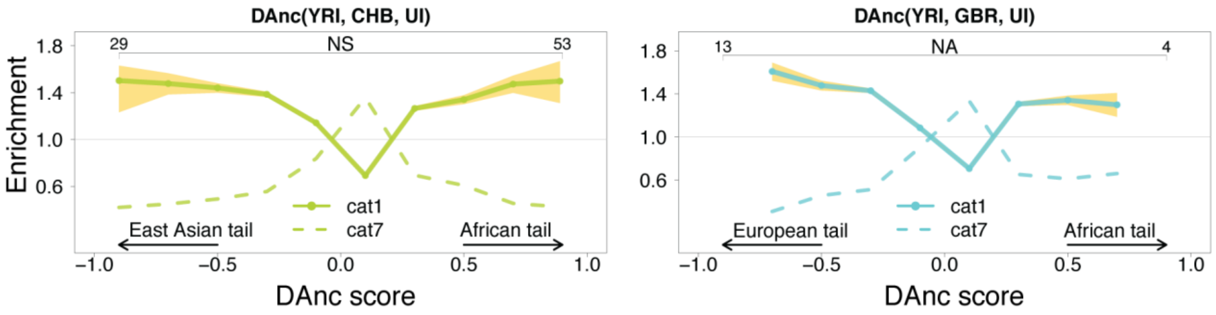

Supplementary Figure 30. Results of the enrichment analysis for DAnc(YRI, CHB, UI) and DAnc(YRI, GBR, UI) using as annotation putatively regulatory sites (regulomeDB category 1) and putatively non-regulatory sites (regulomeDB category 7). The non-regulatory category was downsampled to match the number of variants in the regulatory category (28,452). The bootstrapped 95% confidence interval is shown in yellow, and the level of significance of the bias in regulatory enrichment when comparing the two tails is shown on top (* < 0.05, ** < 0.01, and *** < 0.001, and NS for non-significant). The number of regulatory sites in each tail is on top of the tails. Enrichment is shown per bin only when at least four variants are present in both annotations, CI is calculated regardless.

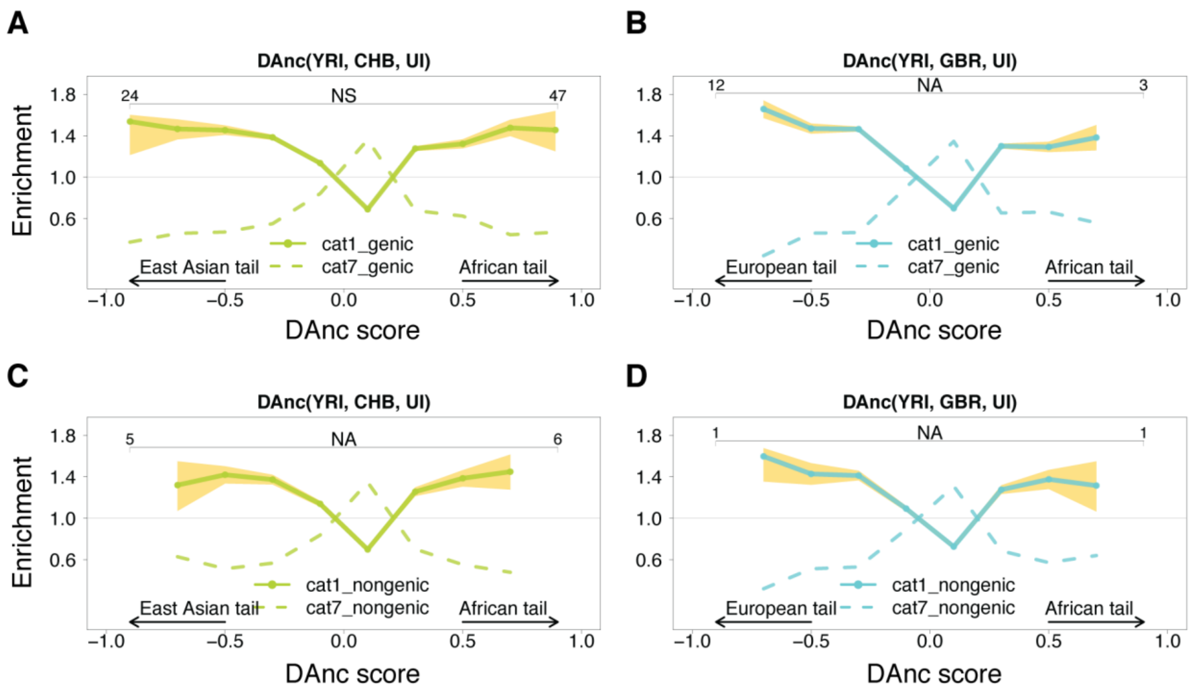

Supplementary Figure 31. Results of the enrichment analysis for (**A** & **C**) DAnc(YRI, CHB, UI) and (**B** & **D**) DAnc(YRI, GBR, UI) using as annotation putatively regulatory sites (regulomeDB category 1) and putatively non-regulatory sites (regulomeDB category 7). The analysis was run separately for variants that are also in the (**A** & **B**) genic category and (**C** & **D**) in the non-genic category of the genic/non-genic annotation. All analyses were downsampled to match the number of variants in the regulatory-genic category (21,957 variants). The bootstrapped 95% confidence interval is shown in yellow, and the level of significance of the bias in regulatory-genic enrichment when comparing the two tails is shown on top (* < 0.05, ** < 0.01, and *** < 0.001, and NS for non-significant). The number of regulatory-genic sites in each tail is on top of the tails. Enrichment is shown per bin only when at least four variants are present in both annotations, CI is calculated regardless.

Supplementary Tables

P1	P2	P3	P4	D value	Z score	BABA[1]	ABBA[2]	# SNPs[3]
NE1	Stuttgart	Loschbour	YRI	0.000101	1.932	25692	25106	5764789
NE1	Stuttgart	CEU	YRI	0.000012	0.574	25694	25623	5764789
NE1	Stuttgart	FIN	YRI	0.000017	0.79	25515	25416	5764789
NE1	Stuttgart	GBR	YRI	0.00001	0.486	25686	25630	5764789
NE1	Stuttgart	TSI	YRI	-0.000015	-0.785	25722	25811	5764789

Supplementary Table 1. D statistics for ancient European genomes and present-day European populations. Linked sites were removed using plink (*--indep-pairwise 200 25 0.4*). A Z score >1.96 or <1.96 indicates significance of the D value on the 95% level.

[1] Describes allele sharing of P1 with P3.

[2] Describes allele sharing of P2 with P3.

[3] Total number of SNPs used.

Set	# SNPs	reduced background	complete background
European tail	497	-	-
Stuttgart specific	37	-	helicase_activity (0.046)
			pyrophosphatase_activity (0.048)
			hydrolase_activity,_acting_on_acid_anhydrides,_in_phosphorus-containing_anhydrides (0.048)
Loschbour specific	55	melanosome_membrane (0.003)	melanosome_membrane (0.004)

Supplementary Table 2. Enrichment of gene ontology categories. Genes intersecting with alleles in the European DAnc (YRI, P_2, Ust'-Ishim) tails (P_2: GBR, FIN, TSI, or CEU) and that are found exclusively in one of the two ancient Europeans (exclusively in Stuttgart or in Loschbour). Results are shown for the reduced background (random 100,000 alleles) and the complete background (all alleles), with P-values in parenthesis.

Supplementary Notes

Supplementary Note 1. DAnc analysis using annotation based on protein-changing, putative regulatory role, or conservation.

Different annotations of the genome-wide data were used in the DAnc analysis. To capture potential different aspects of phenotypic relevant variation we used the following annotations:

- Nonsynonymous / Synonymous
- conserved / non-conserved (phastCons > 0.9 / phastCons < 0.1)
- regulatory / non-regulatory (ENCODE: regulomeDB category 1 / regulomeDB category 7); RegulomeDB[12] incorporates ENCODE and other sources of regulatory information on variants. It classifies variants into seven categories based on the amount of evidence for a regulatory role. Variants in category 1 show multiple evidences for regulatory effects, while variants in category 7 show none.

We performed the DAnc analysis for each annotation using YRI as P_1 and GBR (European) or CHB (East Asian) as P_2,. although we note that the low number of variants makes many of these analyses quite noisy. The regulomeDB and phastCons analyses have very different number of variants in their two categories (conserved 516,448 vs. non-conserved 15,422,612; regulatory 28,452 vs. non-regulatory 3,925,604). To avoid an effecting of these differences the DAnc analysis we downsampled, in each annotation, the category with the larger number of variants to match the category with the lowest number of variants (516,448 for the conservation annotation and 28,452 variants for the regulatory annotation). The downsampling has no impact on our original DAnc analysis (Supplementary Fig. 27).

There is no enrichment in the DAnc tails for the protein-changing annotation (Supplementary Fig. 28, likely due to low number of variants) or for the conservation annotation (Supplementary Fig. 29). Only the regulomeDB DAnc analysis (Supplementary Fig. 30) shows significant enrichment of putatively functional alleles (regulatory variants) over putatively non-functional ones (non-regulatory variants) in the DAnc tails. The enrichment is significant in all tails, even though it lacks sufficient non-functional alleles in both tails of the DAnc(YRI, GBR, UI) analysis. Nevertheless, when we combine both annotations (genic/non-genic with regulomeDB category 1/7) it becomes clear that the enrichment in alleles in the regulatory category is largely due to alleles in the genic annotation (Supplementary Fig. 31), suggesting a regulatory role for the strongly differentiated alleles in the DAnc tails.

Supplementary Note 2. Analyzing a second European Farmer – NE1.

Gamba et al.[13] recently presented the high-coverage genome (22X) of a ~7,000 years old Hungarian Farmer (NE1). This sample was treated differently than Ust'-Ishim, Loschbour and Stuttgart (e.g. it was not UDG treated and it likely contains more ancient DNA damage) and no estimate of autosomal contamination is available. Direct comparisons with the other genomes must thus be cautious, but NE1 allows us to explore again the signatures observed in the Stuttgart farmer.

Dr. Gamba kindly shared the filtered BAM file of NE1, which we used to call genotypes following the methods section in[13]. In genome-wide PCA analysis the Stuttgart and NE1 farmers fall together (Supplementary Fig. 24). Also, D statistics show that the two farmers are equally close to present-day European populations (Supplementary Table 3), indicating a similar contribution from their populations to modern Europeans. Nevertheless when the three ancient samples are considered Loschbour is closer to NE1 than to Stuttgart (Supplementary Table 3), although the 1.93 Z-score is not significant under a significance threshold of 95% (+-1.96). This is consistent with some level of gene flow between the Loschbour and NE1 populations.

When we investigate the genic European DAnc tail NE1 tends to contain, like Stuttgart, fewer alleles from the European tail than Loschbour. But the difference between NE1 and Loschbour is weak and not significant. (Supplementary Fig. 25). In fact, a PCA using only SNPs in the European DAnc tail shows Loschbour being closer to NE1 than to Stuttgart (Supplementary Fig. 24). Together these analyses suggest that although at the genome level the two farmers are very similar, NE1 already contained, perhaps by gene flow from hunter-gatherers, some of the highly differentiated European alleles that are absent in the Stuttgart genome.

Supplementary References

1. Weir, B. S. & Cockerham, C. C. Estimating F-Statistics for the Analysis of Population Structure. *Evolution* **38,** 1358 (1984).
2. Gravel, S., Henn, B. M., Gutenkunst, R. N., Indap, A. R., *et al.* Demographic history and rare allele sharing among human populations. *Proceedings of the National Academy of Sciences* **108,** 11983-11988 (2011).
3. Fu, Q., Li, H., Moorjani, P., Jay, F., *et al.* Genome sequence of a 45,000-year-old modern human from western Siberia. *Nature* **514,** 445-449 (2014).
4. Gravel, S., Henn, B. M., Gutenkunst, R. N., Indap, A. R., *et al.* Demographic history and rare allele sharing among human populations. *Proceedings of the National Academy of Sciences* **108,** 11983-11988 (2011).
5. Lazaridis, I., Patterson, N., Mittnik, A., Renaud, G., *et al.* Ancient human genomes suggest three ancestral populations for present-day Europeans. *Nature* **513,** 409 (2014).
6. Haak, W., Lazaridis, I., Patterson, N., Rohland, N., *et al.* Massive migration from the steppe was a source for Indo-European languages in Europe. *Nature* **522,** 207-211 (2015).
7. Mathieson, I., Lazaridis, I., Rohland, N., Mallick, S., *et al.* Genome-wide patterns of selection in 230 ancient Eurasians. *Nature* (2015).
8. Cavalli-Sforza, L. L. Genetic and Cultural Diversity in Europe. *Journal of Anthropological Research* **53,** 383-404 (1997).
9. Patterson, N., Price, A. L. & Reich, D. Population structure and eigenanalysis. (2006).
10. McVicker, G., Gordon, D., Davis, C. & Green, P. Widespread Genomic Signatures of Natural Selection in Hominid Evolution. *PLoS Genet* **5,** e1000471 (2009).
11. Meyer, M., Kircher, M., Gansauge, M. -T., Li, H., *et al.* A high-coverage genome sequence from an archaic Denisovan individual. *Science* **338,** 222-226 (2012).
12. Boyle, A. P., Hong, E. L., Hariharan, M., Cheng, Y., *et al.* Annotation of functional variation in personal genomes using RegulomeDB. *Genome Research* **22,** 1790-1797 (2012).
13. Gamba, C., Jones, E. R., Teasdale, M. D., McLaughlin, R. L., *et al.* Genome flux and stasis in a five millennium transect of European prehistory. *Nature Communications* **5,** (2014).
